# Transcriptional Changes of the Root-Knot Nematode *Meloidogyne incognita* in Response to *Arabidopsis thaliana* Root Signals

**DOI:** 10.1371/journal.pone.0061259

**Published:** 2013-04-12

**Authors:** Alice Teillet, Katarzyna Dybal, Brian R. Kerry, Anthony J. Miller, Rosane H. C. Curtis, Peter Hedden

**Affiliations:** 1 Rothamsted Research, Harpenden, Herts, United Kingdom; 2 Department of Plant Pathology, University of Wisconsin, Madison, Wisconsin, United States of America; 3 John Innes Centre, Norwich Research Park, Colney, Norwich, United Kingdom; Volcani Center, Israel

## Abstract

Root-knot nematodes are obligate parasites that invade roots and induce the formation of specialized feeding structures. Although physiological and molecular changes inside the root leading to feeding site formation have been studied, very little is known about the molecular events preceding root penetration by nematodes. In order to investigate the influence of root exudates on nematode gene expression before plant invasion and to identify new genes potentially involved in parasitism, sterile root exudates from the model plant *Arabidopsis thaliana* were produced and used to treat *Meloidogyne incognita* pre-parasitic second-stage juveniles. After confirming the activity of *A. thaliana* root exudates (ARE) on *M. incognita* stylet thrusting, six new candidate genes identified by cDNA-AFLP were confirmed by qRT-PCR as being differentially expressed after incubation for one hour with ARE. Using an *in vitro* inoculation method that focuses on the events preceding the root penetration, we show that five of these genes are differentially expressed within hours of nematode exposure to *A. thaliana* roots. We also show that these genes are up-regulated post nematode penetration during migration and feeding site initiation. This study demonstrates that preceding root invasion plant-parasitic nematodes are able to perceive root signals and to respond by changing their behaviour and gene expression.

## Introduction

Plant parasitic nematodes (PPNs) are a major threat to agriculture causing about $100 billion in crop losses annually [1]. The root-knot nematode (RKN) *Meloidogyne incognita* is able to infect almost all cultivated plants [2], including the model plant *Arabidopsis thaliana* [3], [4]. The parasite cycle starts when the pre-parasitic juveniles (J_2_), after hatching from the eggs, invade the root at the elongation zone and migrate towards the root tip where they enter the vascular cylinder. The nematodes then induce the differentiation of 5 to 7 parenchyma cells leading to the development of large multinucleated cells, called giant cells which form the nematode feeding site. Once the feeding site is formed, the J_2_ become sedentary and develop into third (J_3_), fourth (J_4_) stage juveniles and finally into adult females which produce eggs that are extruded at the surface of the root [5].

For survival as an obligate biotrophic pathogen, *M. incognita* relies on its ability to successfully locate and infect its host. It is generally accepted that plant infection is facilitated by effector proteins secreted by PPNs and the identification of candidate effectors has been the subject of a number of studies [6]–[8]. RKN secretions containing these proteins could potentially originate from a variety of different organs, but most of the work has focused on stylet secretions from the oesophageal glands, which consist of one dorsal and two subventral glands. Differential gene expression and cDNA library analysis of the oesophageal glands have been used to identify potential parasitism genes in *Meloidogyne spp*. J_2_ [9], [10] and direct analysis of *M. incognita* secretions via a proteomic approach has recently allowed the identification of nearly 500 secreted proteins [11]. In addition, bioinformatics analysis on assembled ESTs from *M. chitwoodi*, which has also a wide host range among arable crops, predicted 398 putative secreted proteins, among which 8 were confirmed to have their transcripts localized in the oesophageal glands [12]. Besides their putative role during invasion and migration, stylet secretions have also been suggested to induce differentiation of the giant cells. A suppression subtractive hybridization approach between *M. incognita* pre-parasitic J_2_ and J_3_ nematodes extracted from *Arabidopsis thaliana* galls showed that at least 50 genes were up-regulated in the parasitic stage [13].

cDNA-AFLP approaches have also proved to be an efficient strategy for identifying potential parasitism genes, such as those reported for *Globodera rostochiensis* [14]. This method has also been used to compare transcripts between virulent and avirulent strains of RKN. In *M. javanica*, transcriptome comparison of the strains VW4 and VW5 has led to the identification of *Cg-1*, a gene required in the nematode for triggering *Mi-1*-mediated plant resistance [15] while in *M. incognita* 22 transcript-derived fragments (TDFs) have been isolated, among which at least three were localized in the oesophageal glands [16].In addition to these studies, the recent availability of the *M. hapla* and *M. incognita* genome sequences will lead to a better understanding of PPN parasitism genes [17], [18].

Although many nematode-secreted proteins or genes encoding secreted proteins have been identified, it is important to obtain a better understanding of their roles *in planta* during the infection process. Some *M. incognita* proteins are preferentially located in the plant apoplasm throughout migration and feeding site establishment [19]–[21]. In addition, *M. incognita* protein Mi-EFF1 has been recently shown to be secreted from the sedentary nematode within the giant cell and is targeted at the nuclei, where it may manipulate nuclear function of the host cell [22]. It has also been reported that cyst nematode effectors target plant proteins directly in order to reprogram cell functions [23]–[25]. These studies highlight that whilst we have increased our understanding of *in planta* events following infection, little is known about the signalling mechanisms that occur before nematodes penetrate the roots. In fact, genes potentially involved in parasitism have been mainly identified from either pre-parasitic J_2_ that have not been exposed to root tissues or signals [8], or from nematodes that were already inside the roots [13], but no studies have reported changes in J_2_ gene expression preceding the actual penetration of the root.

It is generally acknowledged that root exudates play a major role in the attraction of PPNs to their host roots. Reports of nematode attraction/aggregation [26]–[28] and avoidance/repellence [29], [30] to root exudates have been published. Root exudates also promote nematode hatching, especially for the cyst nematodes [31]. When at the surface of the root, PPNs use their stylet thrusts to weaken the epidermal cell wall and to release secretions [3]. Interestingly, in the absence of roots, potato and legume root diffusates/exudates induce secretion from *G. rostochiensis* and *M. incognita*, respectively, while mustard root exudates induce *Heterodera schachtii* stylet movements [26], [30], [32], [33]. These reports emphasize the role of root exudates on nematodes behaviour, and although these behavioural changes may result from transcriptional changes, the regulation of nematode gene expression by root signals has been studied specifically in relation to the hatching of cyst nematodes [34]–[36]. To our knowledge no nematode genes potentially involved in parasitism have been shown to be regulated by root exudates.

The model plant *A. thaliana* with its wealth of genetic and genomic tools has been widely used to study plant interactions with PPNs, especially to identify plant genes that are differentially regulated upon nematode infection [37]–[40]. To date, crop plants such as tomato or alfalfa have been used in order to study events that happen before root penetration [11], [30]. Here we investigate whether *A. thaliana* root exudates have an effect on *M. incognita* gene expression. After showing that root exudates from *A. thaliana* were active by inducing changes in nematode stylet thrusting activity, we used a cDNA-AFLP approach to identify novel candidate parasitism genes. Six new genes were validated by qRT-PCR as being differentially regulated following exposure to root exudates. In order to confirm that these genes were also differentially expressed when nematodes were in contact with the roots, we used an *in vitro* inoculation method that focuses on the events preceding the nematode penetration of the roots. Using this method we confirmed that the genes responsive to root exudates were also strongly differentially expressed when in contact with root tissue signals (pre-penetration) and after root penetration.

## Results

### Sterile *Arabidopsis thaliana* root exudates induce *Meloidogyne incognita* stylet thrusting activity

**Figure 1:**
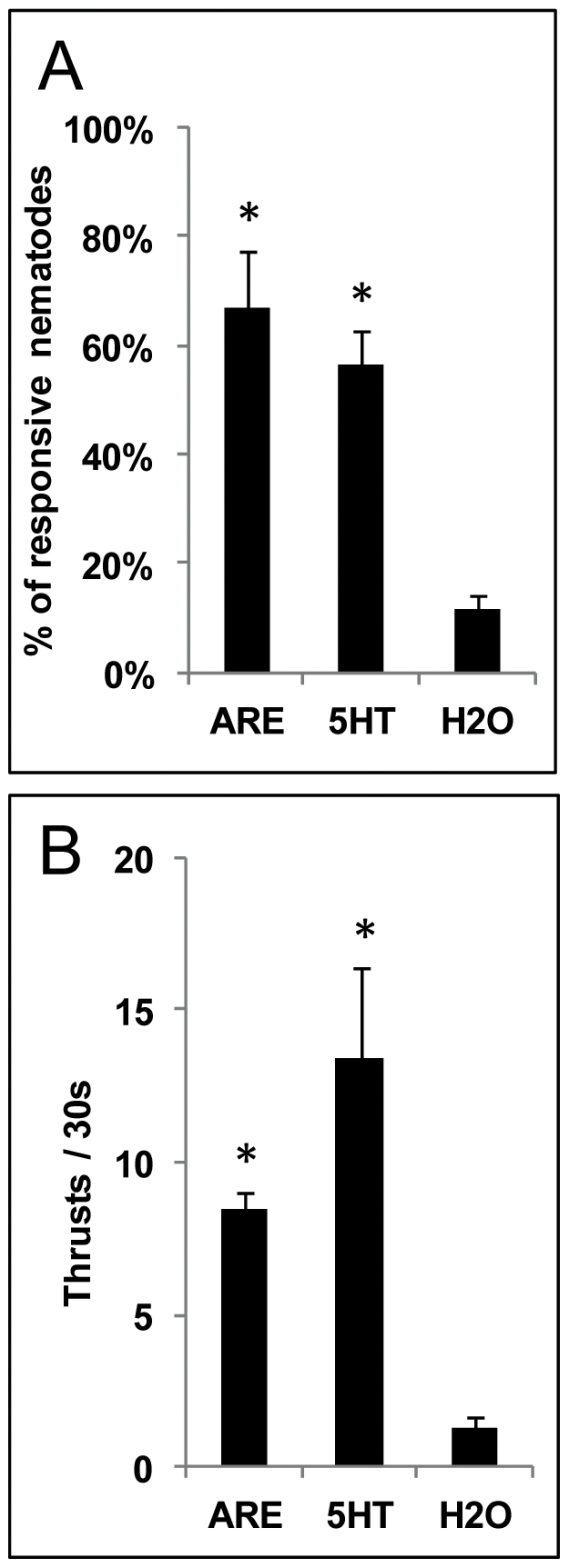
Effect of *A. thaliana* root exudates (ARE) on *M. incognita* stylet movements. **A**, Percentage of responsive nematodes and **B**, frequency of thrusting after 15 min of treatment with 20 µL of ARE (equivalent to 0.45 mg of dry weight of root) (ARE), 5 mM 5-hydroxytryptamine (5HT) or distilled water (H_2_O). Error bars indicate +/− SEM. Statistical significance (ANOVA) of pair-wise comparisons between treatments (ARE or 5HT) and the water control is indicated by asterisks (P<0.05).

Before investigating whether *A. thaliana* root exudates (ARE) were able to trigger changes in *M. incognita* gene expression, we used a behavioural assay to ensure that the nematodes were responsive to ARE. Sterile root exudates were collected from hydroponically grown *A. thaliana* plants and resuspended in water. The response of *M. incognita* pre-parasitic second stage juveniles (J_2_) to ARE was monitored using a simple assay based on their stylet thrusting activity (repulsion/retraction of the stylet). We found that ARE, when concentrated, triggered stylet movements similar to that observed in response to the positive control 5 mM 5-hydroxytryptamine (5HT), which has been shown to trigger stylet thrusting activity [41], [42]. When treated for 15 min with 20 µL of sterile ARE (equivalent to approximately 0.45 mg dry weight of root), 66% of *M. incognita* J_2_ displayed stylet movements at a frequency of 8.5 pulses/30 s, which was comparable to their response to 5 mM 5HT (56% of responsive J_2_, 13.4 pulses/30 s) (Fig. 1A and B). *M. incognita* stylet thrusting activity without stimulation (water control) was significantly different from that observed after treatment with 5HT or ARE (P<0.05), with only 11% of J_2_ responding at 1.3 pulses/30 s.

### Identification of *M. incognita* differentially expressed transcripts in response to ARE by cDNA-AFLP

**Figure 2:**
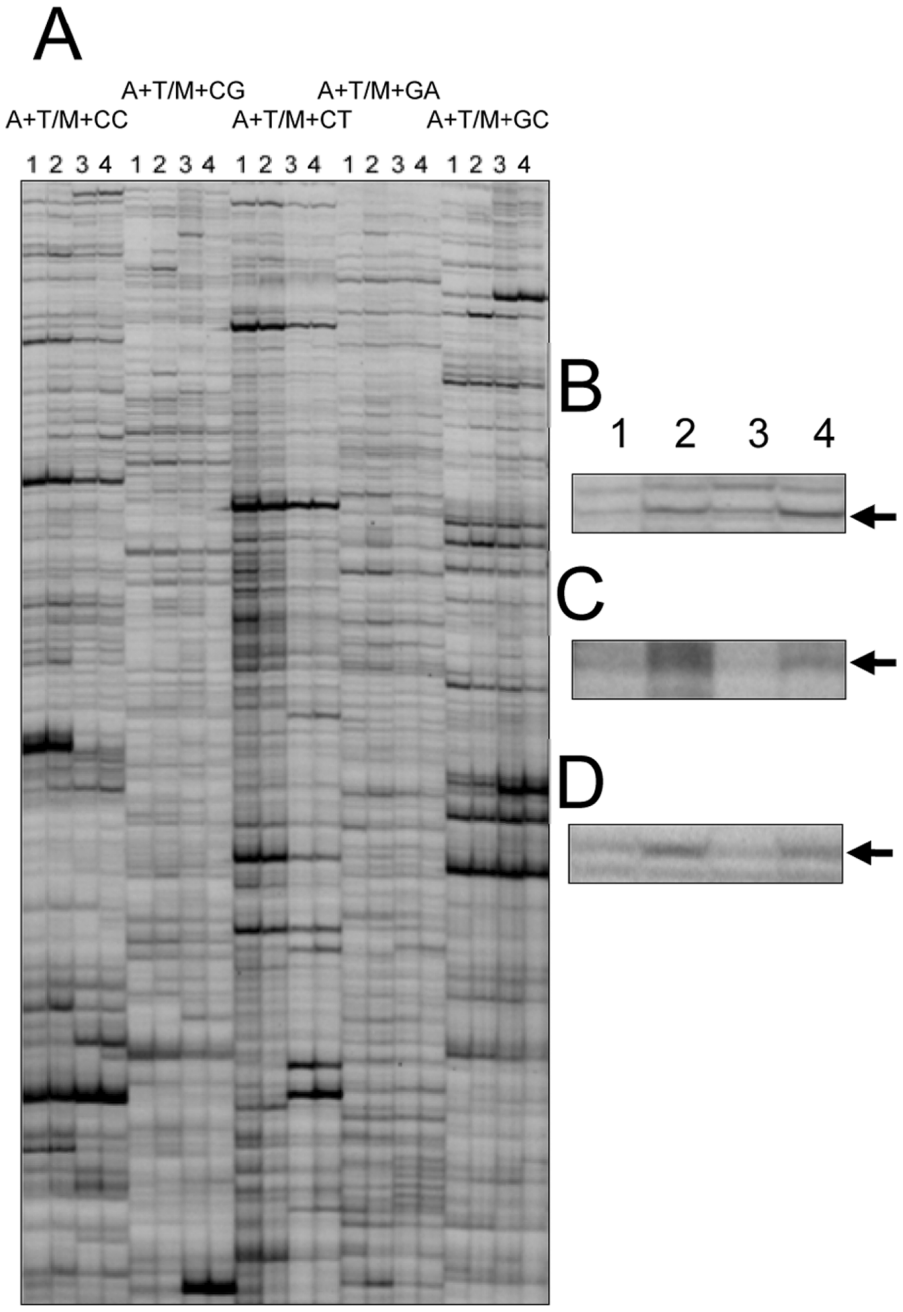
cDNA-amplification fragment length polymorphism patterns from *M. incognita* generated with A+1/M+2 primer combinations. A, Patterns generated with different primer combinations. B through D, selected part of gels showing differential transcript-derived fragments: B, P57E2 obtained with A+T/M+CC; C, P64A1, obtained with A+C/T+CG; and D, P66E1, obtained with A+C/T+GT. Lanes 1, 2, 3 and 4 = cDNA from H_2_O- (1 and 3) and ARE- (2 and 4) treated J_2_ from 2 biological repeats.

To analyse if ARE affect *M. incognita* gene expression, cDNA-AFLP was carried out on J_2_ nematodes treated for 1 h with ARE or water, across two biological repeats. Using two pairs of restriction enzymes *Ase*I/*Mse*I and *Ase*I/*Taq*I, 44 transcript-derived fragments (TDFs) were selected as being more abundant in ARE-treated J_2_ than in those treated with water (see examples in Fig. 2). In addition, 19 TDFs were selected as being less abundant in ARE-treated compared to water-treated J_2_. The 63 differentially expressed TDFs were sequenced (Table S1) and a BLASTN search was performed directly to the *M. incognita* genome sequence or, when no match was found, to the NCBI database. The sequenced TDFs were between 31 to 364 bp long and had between 92 to 100% identity to the matched sequences. Among them, 48 matched predicted *M. incognita* protein-encoding genes, 7 matched *M. incognita* ESTs, 5 matched unplaced reads, whilst 3 matched genomic sequences where no proteins or ESTs were predicted in the genome database (Table S2). Protein or nucleic acid sequences were analysed by BLASTP and BLASTX, respectively, for their sequence similarity to known proteins at the NCBI database. Nearly half of the sequences showed highest homology with genes/proteins from parasitic organisms, most commonly from the animal nematodes *Brugia malayi* and *Ascaris suum* (Table S2).

TDFs corresponding to predicted proteins or ESTs were classified into the 9 protein subfamilies described in Bellafiore *et al* (2008) [11]. Thirty three per cent of the TDFs correspond to predicted proteins of unknown function (family 9), of which 7 displayed no match to sequences in databases. The other TDFs were classified as proteins interacting with actin/microtubules (6TDFs, family 1), or with nucleic acids (8TDFs, family 2), or involved in post-translational modifications, protein turnover and chaperone functions (4TDFs, family 3), metabolism (10 TDFs, family 4), signal transduction (9TDFs, family 5), protein synthesis and secretion (3 TDFs, family 6) and cell wall modification enzymes (2 TDFs, family 8) (Table S2). No TDFs corresponded to proteins predicted to be involved in detoxification.

To broaden the analysis of the 63 TDFs, we used the SignalP and WoLF PSORT software [43], [44] to predict the total number of potential secreted proteins. Ten TDFs correspond to proteins with a predicted signal peptide and/or are predicted to be localized in the extracellular space (Table S2), suggesting a potential role in parasitism.

Interestingly, out of 63, 5 TDFs show sequence similarities to proteins previously shown via proteomics approaches to be secreted during infection and/or when nematodes were treated with tomato root exudates and resorcinol [11], [21], indicating that our transcript based approach is consistent with previous studies. Two of these TDFs correspond to proteins of unknown function (P54E4 and P612E2), one corresponds to a β-1, 4-endoglucanase (P16GH1) while P13GH3 and P512A1 correspond, respectively, to a myosin fragment and a troponin. Interestingly, among these candidates only P16GH1 and P54E4 have a signal peptide and are predicted to be localised in the extracellular space by WoLF PSORT.

### qRT-PCR validation of the TDFs in response to ARE

**Figure 3:**
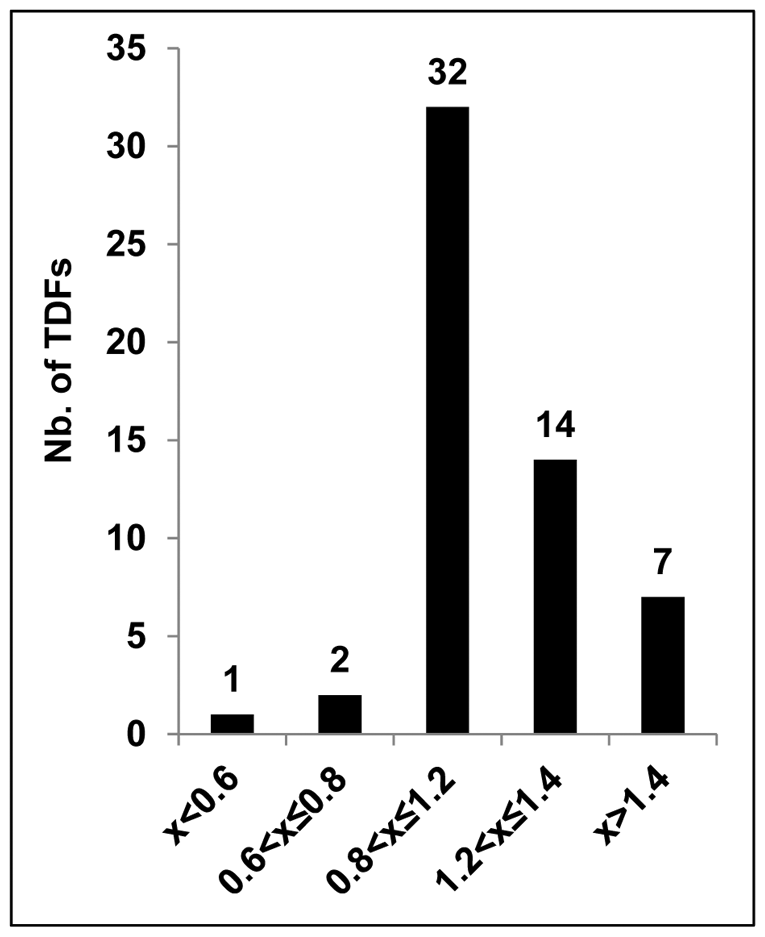
Classification of the 56 TDFs according to their expression levels. The 56 transcript-derived fragments are classified according to the ratio of their expression levels in ARE-treated J_2_ relative to those in water-treated controls (2 biological repeats).

Transcription of the genes corresponding to the 63 selected TDFs was analysed in 2 independent biological repeats by qRT-PCR using mRNA extracted from J_2_ treated for 1 h with ARE or water. When the nucleotide sequence of a TDF was homologous with 2 or more genes, expression levels of each gene were analysed. TDFs were then categorized according to their level of expression in ARE-treated J_2_, normalized against that in water-treated controls (Fig. 3, Table 1 and S2). In total, expression levels of 56 TDFs were determined. Among them 32 TDFs showed no change in gene expression in ARE-treated J_2_ while 3 TDFs were less abundant in ARE-treated J_2_ (Fig. 3). When transcripts corresponding to TDFs were more abundant in ARE-treated nematodes, their levels of expression ranged between 1.2- to 2.89-fold of that measured in the water-treated controls, including 7 with expression levels >1.4 (Fig. 3, Table S2).

**Table 1:** Summary of the 6 transcript-derived fragments (TDFs)^a^

TDF name	*M. incognita* genome or NCBI accession no.	*M. incognita* genome or NCBI annotation	Highest homology in BLASTP or BLASTX	BLASTP or BLASTX E value	Signal peptide^b^	Predicted localization WoLF PSORT^c^	Protein class^d^	Gene expression normalised^e^
**P16AB6**	Minc03819	Ovarian tumour, otubain	Ubiquitin thioesterase otubain-like protein [*Ascaris suum*]	1.E-80	N	Plasma membrane	3	0.84
**P16EF1**	MiV1ctg1025:1.778	Na	hypothetical protein DDB_G0280555 [*Dictyostelium discoideum* AX4]	3.E-02	Na	Na	9	0.52
**P17AB3**	Minc00672	EF-Hand type	calmodulin, putative [*Magnaporthe oryzae* 70–15]	7.E-04	Y	Extracellular	5	0.83
**P57E2**	Minc18288	Not annotated	No homology		Y	Extracellular	9	2.89
**P64A1**	AW828322	Diamine acetyltransferase	hypothetical protein CRE_27333 [*Caenorhabditis remanei*]	1.E-18	N	cyto: 17.5, cyto_nucl: 13.5, nucl: 6.5, cysk: 4.0	4	1.87
**P66E1**	Minc04733	Not annotated	No Homology		N	Extracellular	9	1.66

a Differential expression between ARE- and H_2_O-treated *Meloidogyne incognita* infective juveniles.

b Presence (Y) or absence (N) of signal peptide (SP) was determined by using SignalP software. When no full cDNA or protein sequence was available, the presence of a SP was not determined (ND).

c Putative localisation of the protein corresponding to the TDFs was analysed using WoLF PSORT software. Only 1 predicted localisation was reported when the score was ≥18.

d Proteins were classified by family using the classification published in Bellafiore et al., 2008. (1 =  proteins interacting with actin/microtubules, 2 =  proteins interacting with nucleic acids, 3 =  post-translational modifications, protein turnover, and chaperone functions, 4 =  metabolism, 5 =  signal transduction, 6 =  protein synthesis and secretion, 7 = detoxification, 8 =  cell wall modification enzymes, 9 =  others).

e Gene expression is reported for ARE-treated nematodes in comparison to H_2_O-treated nematodes (2 biological repeats).

**Figure 4:**
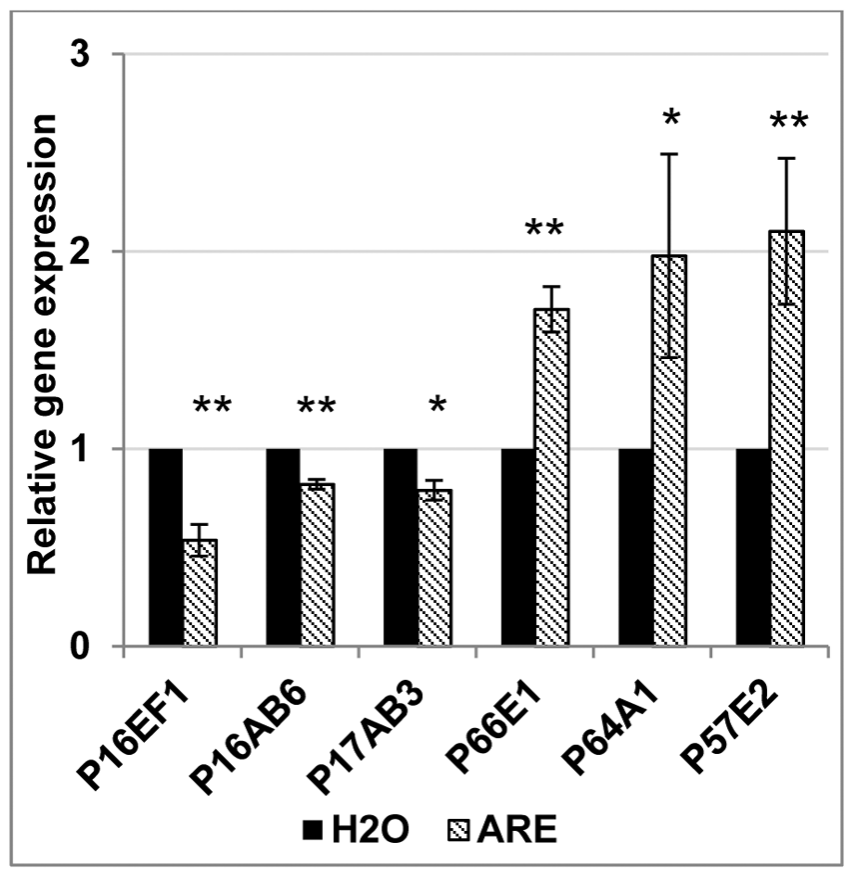
*A. thaliana* root exudates (ARE) regulate *M. incognita* gene expression. Gene expression analysis by qRT-PCR of 6 selected transcript-derived fragments when *M. incognita* juveniles are treated with ARE or water for 1 h. Error bars indicate +/− SEM (3 biological repeats). Statistical significance (ANOVA) of pair-wise comparisons between ARE- and water-treated nematodes is indicated as follows: * = P<0.05, ** =  P<0.01.

We selected 3 TDFs which were consistently down-regulated by ARE (P16EF1, P17AB3 and P16AB6) and 3 TDFs consistently up-regulated (P66E1, P64A1 and P57E2) for further expression analysis. In one further biological repeat (making 3 in total), transcripts of P64A1 and P57E2 were 2 times more abundant in ARE-treated J_2_ than in those treated with water (P<0.05 and P<0.01, respectively), while transcripts of P66E1 were also confirmed to be more abundant in ARE-treated J_2_, but to a lesser extent (P<0.01) (Fig. 4). Conversely, when nematodes were treated with ARE, transcripts for P16EF1, P16AB6 (P<0.01) and P17AB3 (P<0.05) were less abundant than in nematodes treated with water (Fig. 4).These results confirm that ARE induce changes in nematode gene expression in the absence of roots.

TDFs P16AB6, P17AB3, P57E2 and P66E1 correspond, respectively, to predicted *M. incognita* proteins Minc03819, Minc00672, Minc18288 and Minc04733, while P16EF1 corresponds to a genomic sequence supported by ESTs (Table 1). P64A1 corresponds to an EST (AW828322) homologous with *C. elegans* D2023.4 diamine acetyltransferase (Table 1). *Minc03819* codes for a 305 aa protein with a predicted otubain domain between positions 99 and 296 and shows a significant homology with an *Ascaris suum* ubiquitin thioesterase (Table 1).

Minc00672, Minc18288 and Minc04733 are predicted to be secreted proteins by WoLF PSORT, although only Minc00672 and Minc18288 have a predicted signal peptide (Table 1). Minc18288 and Minc04733 have no homologues in NCBI databases (Table 1), but, for both proteins, homologues were found in *M. hapla* (data not shown). In addition, no conserved domains were predicted using PROSITE [45] or Pfam [46]. *Minc00672* codes for a 149 aa protein with 3 predicted EF-hands domains (PROSITE), suggesting a role in calcium binding.

### Assessment of the *M. incognita* - *A. thaliana* interaction prior to the root penetration

**Figure 5:**
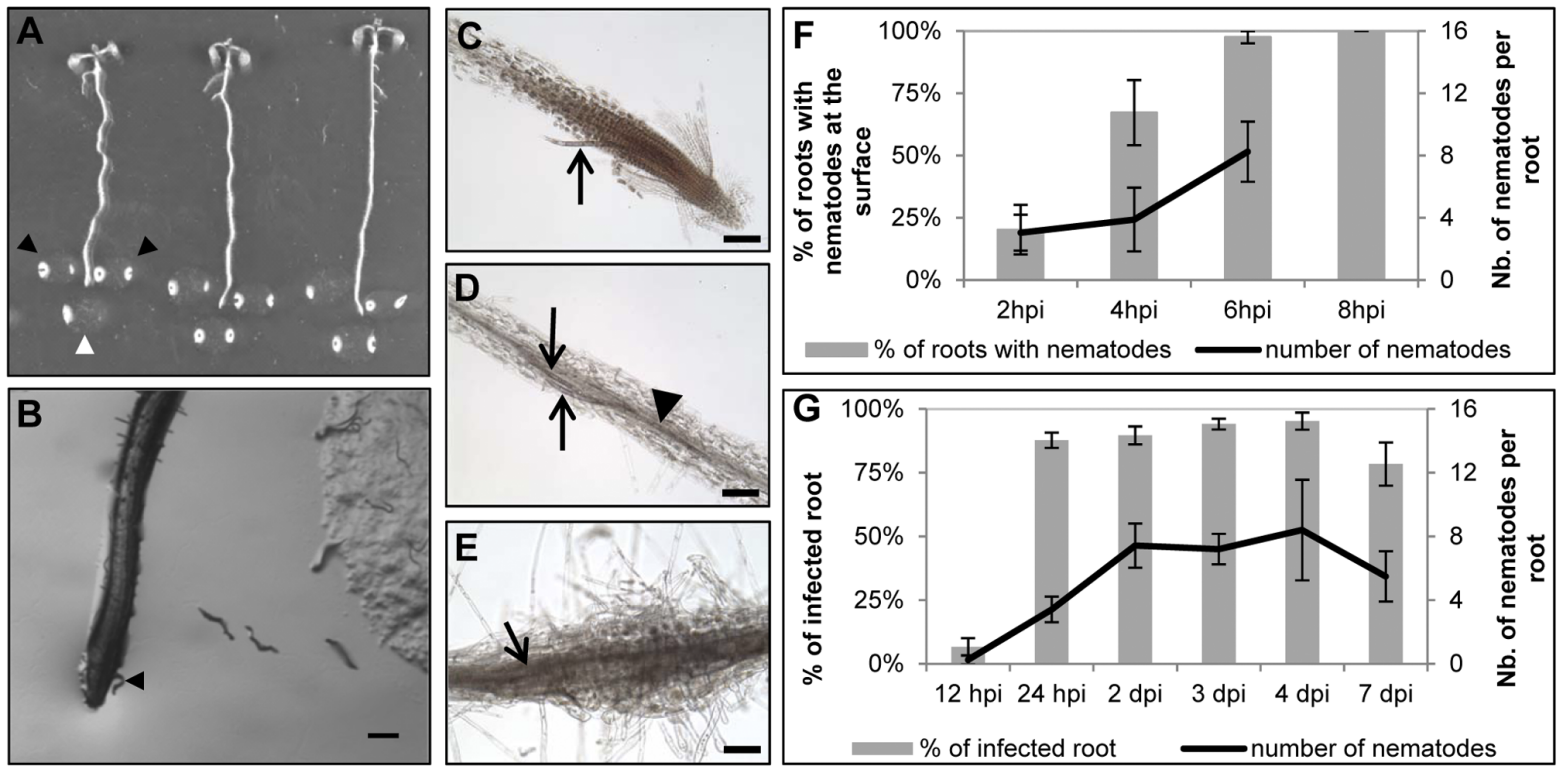
Assessment of the *M. incognita* - *A. thaliana* interaction prior to the root penetration. **A**, *in vitro* inoculation of *A. thaliana* root tips with *M. incognita* infective juveniles in 0.1% agarose (arrowhead) in 3 inoculation points around the root tip. At 8 hours post-inoculation (hpi), the root tip reached the bottom point inoculation (white arrowhead) **B**, Nematode (arrowhead) at the surface of the root tip at 4 hpi, bar = 200 µm. **C** and **D**, Nematodes start to penetrate (arrows) from 12 hpi to 2 days post-inoculation (dpi), while some nematodes could be observed in the vascular cylinder from 2 dpi (arrowhead in **D**). **E**, Initiation of nematode feeding site could be observed at 4 dpi in close proximity to the nematode' head (arrow), bars = 100 µm, **F**, Percentage of roots with nematodes at their surface (grey columns) and number of nematodes present at the root surface (black lines) from 2 to 8 hpi. **G**, Percentage of infected roots (grey columns) and number of nematodes per root (black lines) during penetration, from 12 hpi to 2 dpi and post-penetration, from 2 dpi to 7 dpi.

**Figure 6:**
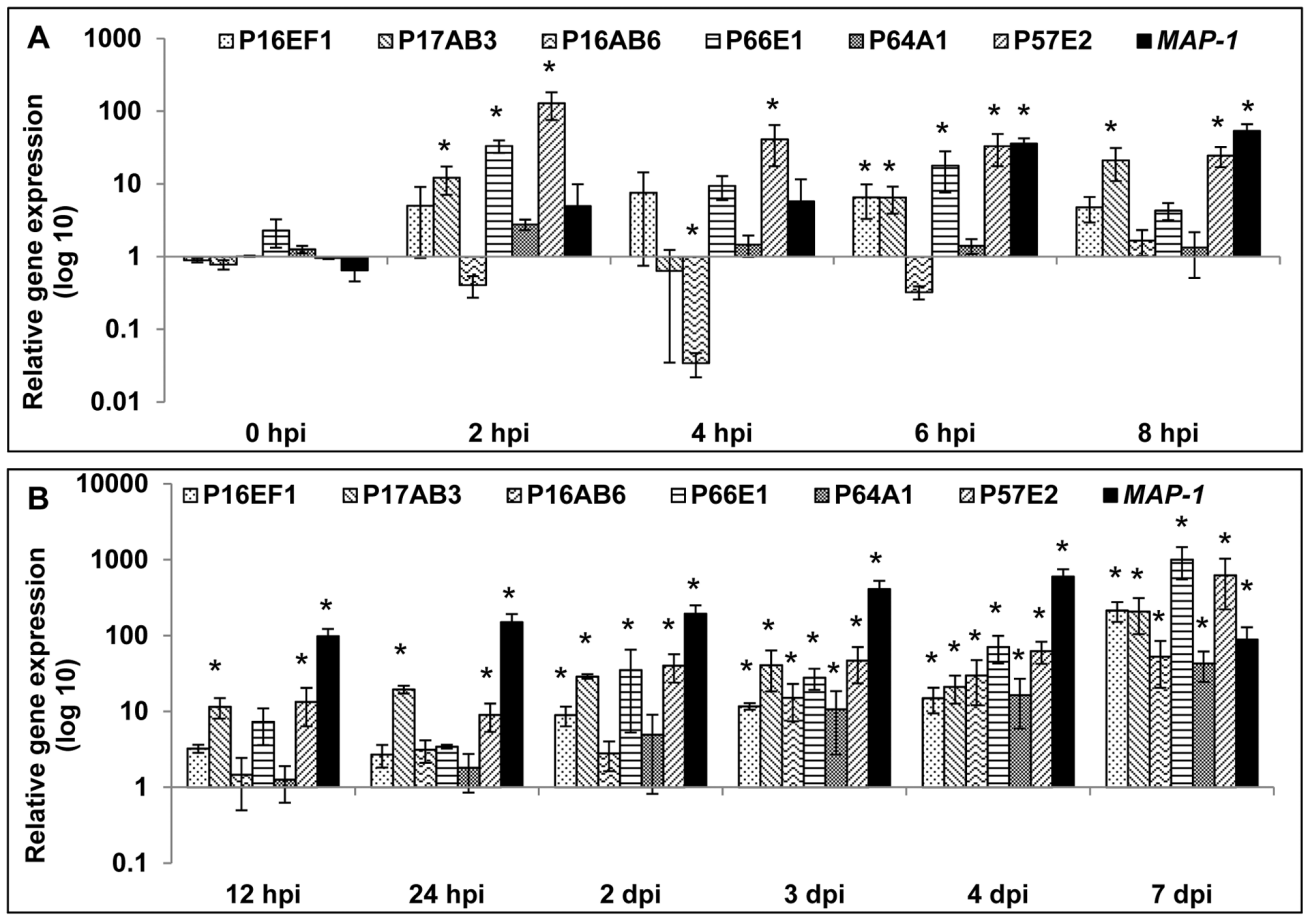
Gene expression analysis of 6 selected TDFs during pre-penetration, penetration and post-penetration stages of the *A. thaliana* - *M. incognita* interaction. A, Gene expression analysis by qRT-PCR of 6 transcript-derived fragments (TDFs) and *MAP-1* during the pre-penetration stage. B, Gene expression analysis by qRT-PCR of 6 TDFs and *MAP-1* during the penetration and the post-penetration stages. Error bars indicate +/− SEM (3 biological repeats). Statistical significance (ANOVA) of pair-wise comparisons between nematodes in close contact with roots (from 2 hpi to 8 hpi), nematodes penetrating or inside the roots (from 12 hpi to 7 dpi) and non-root exposed-nematodes (0 hpi) are indicated by asterisks (P<0.05).

Methods to evaluate *M. incognita* attraction to *A. thaliana* roots [47] and nematode infection followed by the nematode feeding site initiation [3], [4] have been described, but have been often conducted separately. Because this study focused on events preceding root penetration, we used an *in vitro* infection assay to monitor nematode gene expression at this early stage of the interaction. To ensure that our assay was reproducible and synchronised, we first assessed, using a time course infection experiment, nematode attraction and penetration of the root (Fig. 5A to 5F) and then, using the same *in vitro* assay, investigated *M. incognita* gene expression before and after penetration (Fig. 6A and 6B). In these experiments, pre-parasitic nematodes were inoculated 1–2 mm away from the root tip (Fig. 5A and B), allowing the evaluation of nematode attraction to the roots, penetration events and development after penetration.

Under these conditions, we observed nematodes on the surface of the roots from 2 hours post-inoculation (hpi) and at 6 hpi more than 95% of the roots had nematodes at their surface, as shown in Figure 5F. At 8 hpi, roots have grown enough to reach the bottom inoculation point (Fig. 5A), and therefore at that time 100% of roots had nematodes at their surface (Fig. 5F). At 2 hpi an average of 3.04 (+/−1.16) nematodes per root were observed, increasing to 8.24 (+/−1.94) at 6 hpi (Fig. 5F).

To ensure that this assay would also allow a normal infection and development of the nematodes we checked the percentage of infected roots as well as the number of nematodes per root, by collecting and staining the roots from 6 to 24 hpi at 2 h intervals. First penetration events were observed at 12 hpi (Fig. 5C and G) while at 24 hpi more than 80% of the roots were infected with nematodes, with an average of 3.4 +/−0.8 nematodes per infected root (Fig. 5G). At 2 and 3 days post inoculation (dpi), we could observe nematodes inside the vascular cylinder (Fig. 5D), and by 4 dpi initiation of feeding sites was apparent from the swelling of the root (Fig. 5E).

### *M. incognita* genes responsive to root exudates are also differentially expressed before and after penetration of *A. thaliana* roots

Because hydroponically-produced ARE induce changes in *M. Incognita* gene expression, we investigated if these changes were similar to those occurring when J2 were at the root surface. To compare our candidate genes with genes already described, we used as a positive control, *MAP-1*, a gene coding for a protein secreted from *M. incognita* amphidial glands [48] and recently shown to be secreted *in planta* [21]. Study of nematode gene expression during pre-penetration, from 2 to 8 hpi, showed that 4 out of 6 of the candidate genes were up-regulated and one was down-regulated (Fig. 6A). The most highly induced nematode genes were P57E2 and P66E1 at 2 hpi, with, respectively, 128 and 33 times induction in comparison with non-treated nematodes (0 hpi). Although induction of *MAP-1* was not significantly different at 2 and 4 hpi, we could observe a strong induction at 6 and 8 hpi, with respectively 35 and 53 times induction, in comparison with 0 hpi (P<0.05) (Fig. 6A). Up-regulation of P57E2 is consistent with what was observed in ARE treatment, although the induction was stronger. Interestingly, expression of P16AB6 decreased by 97% at 4 hpi (P<0.05) (Fig. 6A), confirming the down-regulation observed in ARE-treated nematodes (Fig. 4). For 3 out of 6 genes, expression patterns were consistent with what was observed with ARE-treated nematodes, confirming that root signals induce nematode gene expression. However the up-regulation of P16EF1 (6 hpi) and P17AB3 (at 2, 6 and 8 hpi) during the pre-penetration stage was in contrast to what was observed in ARE-treated nematodes. Expression of P64A1 did not change significantly during the pre-penetration stage (Fig. 6A).

Analysis of nematode gene expression during the post-penetration stage showed that all the candidate genes, as well as *MAP-1*, were continuously up-regulated in the J_2_ parasitic stage (Fig. 6B). The highest levels of gene expression were at 7 dpi for P57E2 and P66E1, reaching, respectively, 627- and 1008-fold induction in comparison with non-treated nematodes (0 hpi) (P<0.05) (Fig. 6B).

## Discussion

Nematode secretions are thought to play a major role during the plant parasitic interaction by facilitating penetration, dampening plant defences and inducing the formation of giant cells [8], [49]–[51]. The identification of the genes coding for secreted proteins that may function as effectors in these processes has been the focus of a number of studies on RKN and cyst nematodes [6]–[8], [51]. However, although our understanding of the molecular events occurring during penetration and infection is increasing, there is still little known about pre-penetration events.

In this study, sterile root exudates from the model plant *A. thaliana* were produced in order to evaluate their ability to affect gene expression of *M. incognita* pre-parasitic juveniles. Using a stylet thrusting assay we showed that ARE are able to induce nematode stylet movements similar to those observed when nematodes are at the surface of *A. thaliana* roots [3]. This indicates that ARE contain active compounds that are functionally equivalent to the plant signals present at the surface of the roots. Similar stylet movements have been reported for the beet cyst nematode *Heterodera schachtii* in response to mustard root exudates [26]. Root exudates are also able to trigger plant-parasitic nematode protein secretions, as reported for the potato cyst nematode *Globodera rostochiensis* when in contact with potato root diffusates [32], and for *M. incognita* in response to legume root exudates [30]. Tomato root exudates have been used to study *M. incognita* surface cuticle changes [52] and protein secretions when combined with resorcinol [11]; however the effect of tomato root exudates on nematode gene expression has not been reported. To date, it remains unclear whether nematode protein secretion is the result of a release through the stylet of proteins already present in the oesophageal glands or of *de novo* production of proteins from up-regulated transcripts which are induced by plant signals. The present study aimed to investigate the response of RKN to plant signals prior to physical contact and penetration of the root. To test if ARE were able to affect *M. incognita* gene expression, a cDNA-AFLP approach was carried out. Out of 63 potential candidates, 6 candidates were selected for more detailed analysis via qRT-PCR based on consistent gene expression changes. P16EF1, P17AB3 and P16AB6 were consistently down-regulated whilst P66E1, P64A1 and P57E2 were up-regulated within 1 h of ARE treatment, providing the first evidence that root exudates induce changes in *M. incognita* gene expression.

To provide further support for the idea that these changes were in response to plant signals which may be naturally present in the rhizosphere, we used an *in vitro* inoculation method using *A. thaliana* plants on agar plates. Because of their small size and transparency, *A. thaliana* roots are useful materials for studying nematode infection [3], [4], and have been recently used for the *in planta* localisation of *M. incognita*-secreted proteins and to study plant gene expression in response to RKN infection [21], [37]–[39]. In addition to these infection studies, *A. thaliana* seedlings could be used to study pre-penetration events, such as nematode attraction to the root [47]. Our root infection protocol was modified in order to allow the evaluation of events that precede root penetration and to relate them to nematode gene expression across a detailed time-course. Using this procedure, expression of 3 genes (P16AB6, P66E1 and P57E2) during pre-penetration of the roots was confirmed to be consistent with expression patterns observed in ARE-treated nematodes, indicating that ARE could mimic, or contain, plant signals perceived by the nematodes. It is interesting to note that the levels of up-regulation (P66E1 and P57E2) or down-regulation (P16AB6) were stronger in nematodes in contact with root tissues than in ARE-treated nematodes. This could be due to the concentration of ARE or the time of treatment used for these experiments. However, preliminary results indicated that a higher concentration (equivalent to the one used for stylet thrusting assay) or a longer treatment (4 hours) produced a similar pattern and level of gene expression to those observed with our standard conditions (data not shown). Differences in gene expression levels could also be a consequence of disparities in chemical composition of ARE compared with the signals present at the surface of the roots. This might explain the differences in the pattern of expression between nematodes treated with ARE and those near the root surface which were observed for P16EF1 and P17AB3, and it has been suggested that depending on the collection method, root exudates can induce different behavioural responses of RKN [30].

Although differences in gene expression patterns were observed among the six candidates during pre-penetration, post-penetration gene expression data demonstrated an up-regulation of all the genes from 3 dpi onwards. In *M. incognita*, 57 genes mainly involved in detoxification and protein degradation have been reported to be up-regulated in J_3_ stage nematodes extracted from infected *A. thaliana* roots [13]. With the exception of P16AB6, corresponding to *Minc03819*, encoding a putative ubiquitin thioesterase, none of the six selected genes described in this study seems to be involved in protein degradation or in detoxification, rather they have largely unknown function, but some contain signal peptides suggesting that they are secreted (Table 1).

To our knowledge this is the first time that *M. incognita* gene expression has been analysed at such early time points of the interaction, before the actual penetration of the roots (from 2 hpi to 12 hpi). *M. incognita* developmental gene expression is often reported for eggs, pre-parasitic J_2_ (before invasion of the plant, but not exposed to host roots or plant-derived signals), and for parasitic stages (migratory J_2_, J_3_, J_4_ or female) using *in situ* hybridization [11], [53] and/or RT-PCR [16], [54], [55] or qRT-PCR [13], [56]. In laboratory conditions, freshly hatched nematodes are used for the pre-parasitic stage, and in the case of RKN this is in the absence of root exudates as they generally hatch in water [5]. Our data suggest that the transition from the pre-parasitic to the parasitic stage is gradual and associated with various temporally regulated changes in nematode gene expression.

Because of the wide host range of *M. Incognita* it is difficult to establish whether the nematodes recognise specific host signals or more common signals present in root exudates of a broad range of plant species. Some plants have been described to be resistant to *M. incognita*, although nematode penetration of the root tissues is still observed [57]. Similarly, root knot nematode juveniles are able to penetrate roots of resistant tomato plants carrying the *Mi-1* gene [58]. In both examples the plant resistance is characterised by the inability of the nematode to complete its life cycle, but not to locate and penetrate the roots, indicating that root exudates are probably not the key factors for defining the host resistance/susceptibility status of a plant. Instead based on our data, it seems that *M. incognita* is able to recognise signals present in root exudates that trigger a change in gene expression in the nematode juveniles. However, comparing *M. Incognita* transcript profiles in response to root exudates from different plant species could lead to a better understanding of the nature of the signals responsible for these changes. Whether any of the six genes we have identified are required for nematode pathogenicity remains to be determined, by using available methods such as RNA interference to silence the nematode genes [59]. However three of the genes described in this study (P57E2, P17AB3 and P66E1) are up-regulated as early as 2 hpi and their corresponding proteins are predicted to be secreted, although immunolocalisation experiments would be required to confirm their secretion. We also demonstrated that the expression patterns of these genes are similar to that of *MAP-1* which is highly expressed from 6 hpi onwards, especially after penetration of the roots at 12 hpi. *In planta*, MAP-1, an amphidial protein [48], has been shown to be strongly secreted in the apoplasm during *A. thaliana* root invasion and early sedentary stage [21], which is consistent with its gene expression described here.

It is also interesting to notice the up-regulation of P17AB3 during all the studied stages of the interaction (except at 4 hpi). P17AB3 corresponds to the gene *MINC00672* which codes for a protein containing three EF-hand domains suggesting a role in calcium binding. Similarly, Mi-CRT, a calcium binding protein called calreticulin, has been shown to be secreted *in planta* by *M. incognita* during the nematode migration and development of the feeding site [60]. In addition, the use of a calcium channel blocker seems to prevent root invasion by the potato cyst nematode *G. rostochiensis* [61]. Although these studies highlight a potential role for calcium during nematode parasitism it is unclear whether calcium is part of a molecular dialogue between the nematode and the plant, as it has been suggested for other pathogenic or symbiotic interactions [62]. Interestingly, the involvement of calcium during plant-nematode interaction is also supported by the up-regulation of several *A. thaliana* genes encoding calcium transporters in *M. incognita* infested roots [63].

Molecular dialogue between plant and microorganisms has been described for various symbiotic and parasitic interactions. Plant root signals such as strigolactones and flavonoids promote arbuscular mycorrhizal and rhizobia symbiosis, respectively [64]. Plant phenolic compounds have been suggested to be sensed by the necrotrophic fungus *Cochliobolus heterotrophus* and the opportunistic bacterial pathogen *Dickeya dadantii* and to induce the expression of genes involved in parasitism [65], [66]. Interestingly, it has been shown that *M. incognita* produces a factor called NemF which has similar activity on the host root hairs as the Nod Factors produced by rhizobia [67]. The present study shows that a complex molecular communication occurs at the early stages of plant-nematode interactions associated with a number of nematode genes being up- or down-regulated in response to signals present in the root exudates or on the root surface. Identifying the precise plant signals involved in these responses, and determining whether they are required for full nematode pathogenicity, could provide routes to additional novel control strategies. Moreover beyond the scope of this original study we have established a good basis for a linear root infection assay that can now be used to study global changes to the transcriptomes of both *M. incognita* and the host plant *A. thaliana* during infection, for example, by making use of next generation RNA sequencing technologies coupled with the availability of sequenced genomes for both nematode and plant.

## Materials and Methods

### Nematode culture

*Meloidogyne incognita* race 1 NCSU was propagated from greenhouse grown tomato plants (*Lycopersicon esculentum* cv Tiny Tim). After 8 weeks of infection, eggs were recovered from tomato plants by shaking *M. incognita* infected roots in 1∶9 dilution of bleach for 3 min in a flask and rinsed with tap water [68]. Eggs were collected onto 10 µm mesh and were hatched in distilled water at room temperature. When used for infection, second-stage juveniles were surface sterilized using a modified method from Hamamouch et al. (2011) [37]. J_2_ were incubated for 10 min in the sterilisation solution (0.01% mercuric chloride, 0.002% sodium azide and 0.001% Triton X-100), rinsed 3 times with sterile ddH2O and resuspended in sterile agarose 0.1%.

### Plant growth conditions

For hydroponic culture and plate culture, seeds were sterilized in a 20% bleach/0.02% tween solution for 10 min and rinsed 4 times in sterile distilled water. Plant hydroponic culture was modified from a previous method [69]: Autoclaved microcentrifuge 0.5 mL tubes (lid and bottom cut off) were placed in a 1-mL tip rack holder (Starlab,UK), sealed with autoclave tape and autoclaved again. Then under a flow cabinet, tubes were filled from the bottom with 0.5× Murashige and Skoog [70] (MS) (Sigma, UK)/0.8% agar medium, let dry and placed back in the tip box containing 0.5× MS liquid medium. With one sterile *A. thaliana* seed in each tube (60 plants/box), boxes were closed and 6 to 8 boxes were incubated 2 days at 4°C and then transferred to a growth room (12 h day/12 h night, 20°C). After 4 weeks, roots of at least 20 cm were visible under the tubes. Roots from 160 plants (or 2 boxes) were washed for 1 h in sterile distilled water and then root exudates were collected for 24 h in 400–500 mL of sterile distilled water in a shallow glass baking tray. After 24 h, the roots were replaced with roots from 2 other boxes and this procedure was repeated until all the roots were used. At the end of the collection (72 to 96 h), root exudates were placed in 50 mL tubes (Greiner Bio-one limited, UK) and frozen at −20°C. Roots were cut and collected in a 1.5 mL microcentrifuge tube and frozen. Both roots and root exudates were freeze-dried. Root exudates were resuspended in sterile distilled water at a concentration of 45 µL of RE/mg of dry weight roots, aliquoted and stored at −20°C.

For infection experiments, sterile *A. thaliana* seeds were sown on 12 cm square Petri dishes with 0.5× MS medium, 1% sucrose, 0.8% agar and incubated for 48 h at 4°C, then transferred in a growth cabinet (25°C, 16 h light/8 h dark conditions). Four d-old seedlings were then transferred to a new square Petri dish with 0.5× MS medium, 1% sucrose, 0.8% agar (8 plants per plate), before being inoculated with nematodes 24 h later.

### Stylet thrusting assay

Five microlitres of freshly hatched J_2_ (100–200 J_2_) were transferred into 0.5-mL microcentrifuge tubes containing 20 µL of either ddH_2_O (negative control) or test solutions: ARE or 6.25 mM 5-hydroxytryptamine (Sigma, UK). Water was used as a negative control as ARE and the stock solution of 25 mM 5HT were collected or prepared, respectively, in sterile distilled water. Tubes were incubated for 15 min at room temperature prior to observations. The final 25 µL were placed on a glass microscope slide and observed at 200× magnification. Fifteen nematodes were individually observed for 30 s, and the number of thrusts was recorded. Stylet activity of nematodes treated with ARE has been repeated with at least 3 different batches of ARE.

### Nematodes treatment and RNA isolation

For the cDNA-AFLP procedure 300 µL of freshly hatched nematode juveniles (approx. 60 000 J_2_) were incubated with 100 µL of ddH_2_O or ARE in a 12 well plate at room temperature for 1 h. For the gene expression analyses, 20000 J_2_ in 300 µL were incubated in 100 µL of ddH_2_O or ARE in a 12 well plate for 1 h. In both experiments, nematodes were spin down for 2 min at 10000 g and the pelleted nematodes were snap frozen in liquid nitrogen. Nematodes were then ground using a mortar and pestle and total RNA was extracted using the plant RNeasy kit (Qiagen, UK).

### cDNA-AFLP procedure and TDFs sequencing

The transcript content between water and ARE treated J_2_ were compared by cDNA-AFLP as described by Bachem et al. (1996) [71]. First and double strand cDNA were synthesized from 1 µg of total RNA. Double stranded cDNA was digested by two pairs of restriction endonucleases: *Asi*I/*Mse*I and *Ase*I/*Taq*I. After digestion, the restriction fragments were ligated with their corresponding adaptors and pre-amplification was carried out for 25 cycles (94°C, 40 s; 56°C, 60 s; 72°C, 60 s) using primer without selective nucleotide (A+0: 5′-GTAGACTGCGTACCTAAT-3′; M+0: 5′-GATGAGTCCTGAGTAA-3′; T+0: 5′-GATGAGTCCTGACCGA-3′). The PCR products were diluted (10×) in ddH_2_O, and 2.5 µL were used for selective amplification with one selective base extension at the 3′ end of the A+0 primer and 2 selective bases extension at the 3′ end of M+0 and T+0, using a standard AFLP touchdown amplification. The PCR products were separated by electrophoresis in 4.5% denaturing polyacrylamide gels. The gels were dried on 3 MM paper under vacuum and were exposed to Kodak Biomax film.

Bands of interest were cut from the dried gel, soaked in 50 µL of TE buffer and incubated at 4°C for 48 h. PCR with the same selective primers as for the cDNA-AFLP was carried out using 15 µL of the band diffusate. Purified PCR products were then sequenced by Eurofins WMG Operon (Germany). TDFs sequences were compared to the *M. incognita* genome (http://www.inra.fr/meloidogyne_incognita) using the BLAST search. When no hits were found, sequences were compared to the *M. hapla* genome (http://www.pngg.org/cbnp/index.php). All corresponding protein or ESTs sequences were then compared against all sequences in the non-redundant databases, using the BLASTP and BLASTX algorithm on NCBI.

### Nematode inoculation and material collection for staining and gene expression

Twenty microliters of surface sterilised nematodes in agarose 0.1% (approx. 100 J_2_) were inoculated in 3 spots around the root tip as shown on Fig. 5A. Control plants were inoculated in the same conditions with agarose 0.1% only. Thirty-two plants were inoculated per time point and per treatment (with or without nematodes).

To evaluate nematodes attraction to the roots, 16 plants were observed under an inverted microscope (Olympus IMT-2) at 40× magnification. The number of nematodes reaching the roots as well as the number of roots with nematodes at their surface was counted at 2, 4 and 6 hpi. Because the root tips reached the bottom inoculation point (as shown of Fig. 5A) at 8 hpi, all the roots had nematodes at their surface. At that time point, the number of nematodes at the root surface was hard to evaluate precisely as it often exceeded 20.

To evaluate nematodes penetration, 16 plants were collected every 2 h from 6 hpi to 12 hpi and then at 24 hpi, 2 dpi, 3 dpi, 4 dpi and 7 dpi. Plants were stained using a modified acid fuchsin method [72]. Each plant was placed in a well of a 48 well-plate and incubated in 1% bleach for 10 min (15 min for plants collected at 3 dpi onwards). After 2 quick rinses in H_2_O, plants were incubated for 10 min in 1% acetic acid before being transferred into a well of a PCR 96 well plates containing acid fuchsin [72]. The plate was then placed in boiling water for 3 min and plants were then transferred into fresh water until microscopic observations.

For RNA extraction, roots of 16 plants were cut 1 cm above the position of the root tip at inoculation. Surface sterilised pre-parasitic nematodes resuspended in 0.1% sterile agarose were used as the experiments control (0 hpi). For 2 hpi samples, a slice of agar (approx. 1–2 mm around the root, but being careful to not take nematodes remaining inside the area of the inoculation point) was cut in order to collect nematodes surrounding the root; for the other time points just the roots were collected, taking with them the nematodes present at the surface or inside the roots. On average, 10 nematodes per root were collected, giving more than 100 nematodes per time point. Samples were frozen in liquid nitrogen, ground using TissueLyser (QIAgen, UK) and total RNA was extracted using the plant RNeasy kit (QIAgen, UK).

### Gene expression analysis by quantitative RT-PCR

Gene expression analysis was assessed using the method described by Hewezi et al., 2010 [24]. Briefly, total RNA (200–500 ng) was reverse-transcribed using oligo d(T) primers (Invitrogen, UK) and the SuperScriptII Reverse Transcriptase (Invitrogen,UK). RT-qPCR was performed on an Applied Biosystems 7500 with 7500 system SDS software as follows: 95°C for 2 min, followed by 50 cycles of 95°C for 15 s, 60°C for 30 s and 72°C for 45 s, using SYBR® Green JumpStart™ Taq ReadyMix™ (Sigma, UK). Dissociation melting-curve analyses, in which all products generated during the qPCR amplification reaction were melted at 95°C for 1 min, annealed at 60°C for 1 min, and subjected to gradual increases in temperature, were conducted to discount the effects of primer–dimer formation and contamination. Efficiencies of the PCR reactions were determined using the LinReg software (v.12.18) [73]. All primer pairs had efficiencies higher than 1.80. Relative expression of the different gene transcripts was calculated by the delta-delta-C_T_ (ΔΔC_T_) method and converted to the relative expression ratio (2-*ΔΔ*C_T_). All data were normalized to the *M. incognita* endogenous reference gene actin (*MINC06773a*). When using other endogenous reference genes such as the translation elongation factor 1 gene (EF1, *MINC16442*) and the glyceraldehyde 3-phosphate dehydrogenase gene (GPDH, *MINC10963a*) gene expression patterns were similar to those observed using actin (Fig. S1). The gene specific primers used for qRT-PCR are listed in Table S3. Following analysis of variance (ANOVA), least significant differences (LSD) were used to statistically separate the means *Δ*C_T_ (3 biological repeats) at the 5% level of significance (Table S4).

## Supporting Information

Figure S1 **Gene expression analysis of** ***MAP-1*** **and 2 selected TDFs during the** ***A. thaliana*** **-** ***M. icognita*** **interaction using different endogenous gene: Gene expression analysis by qRT-PCR of A,** ***MAP-1*** **, B, P57E2 and C, P16AB6 during the pathogenic interaction (1 biological repeat) using 3 different endogenous reference genes: actin, elongation factor 1 (EF1) and glyceraldehyde 3-phosphate dehydrogenase (GPDH).**
(TIF)

Table S1 **Transcript-derived fragments sequences.**
(DOCX)

Table S2 **Summary of the 63 transcript-derived fragments (TDF)^a^.** ^a^ Differential expression between ARE- and H_2_O-treated *Meloidogyne incognita* infective juveniles ^b^ Presence (Y) or absence (N) of signal peptide (SP) was determined by using SignalP software. When no full cDNA or protein sequence was available, the presence of a SP was not determined (Na). ^c^ Putative localisation of the protein corresponding to the TDF was analysed using WoLF PSORT software. Only 1 predicted localisation was reported when the score was superior at 18. ^d^ Proteins were classified by family using the classification published in Bellafiore et al., 2008. (1 =  proteins interacting with actin/microtubules, 2 =  proteins interacting with nucleic acids, 3 =  post-translational modifications, protein turnover, and chaperone functions, 4 =  metabolism, 5 =  signal transduction, 6 =  proteins synthesis and secretion, 7 = detoxification, 8 =  cell wall modification enzymes, 9 =  others) ^e^ Gene expression is reported for ARE-treated nematodes in comparison to H_2_O-treated nematodes (2 biological repeats).
(DOCX)

Table S3 **Oligonucleotides used for qRT-PCR.**
(DOCX)

Table S4 **Mean ΔCT for statistical comparison.** LSD  =  Least significant difference, degree of freedom (df)  = 19 except for P16EF1 and P17AB3 (df = 18)
(DOCX)
