# Supplementary figures and images for: Transcriptional Changes of the Root-Knot Nematode Meloidogyne incognita in Response to Arabidopsis thaliana Root Signals
Source: PLoS One. 2013 Apr 12;8(4):e61259. doi: 10.1371/journal.pone.0061259 (PMC3625231; doi:10.1371/journal.pone.0061259)

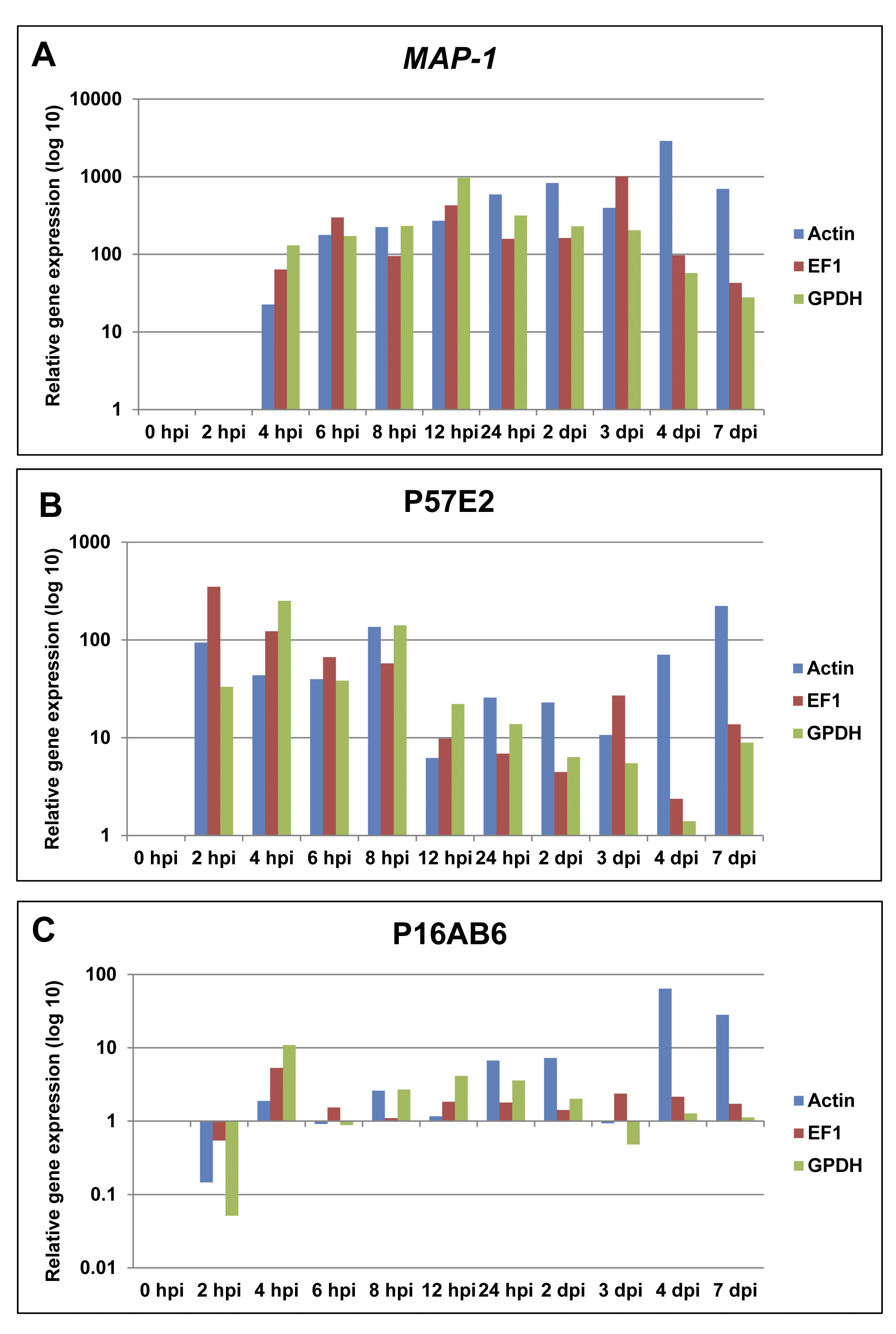

Supplement: Figure S1 — Gene expression analysis of MAP-1 and 2 selected TDFs during the A. thaliana - M. icognita interaction using different endogenous gene: Gene expression analysis by qRT-PCR of A, MAP-1 , B, P57E2 and C, P16AB6 during the pathogenic interaction (1 biological repeat) using 3 different endogenous reference genes: actin, elongation factor 1 (EF1) and glyceraldehyde 3-phosphate dehydrogenase (GPDH). (TIF) [file pone.0061259.s001.tif]
