# Supplementary material for: Transcriptional Changes of the Root-Knot Nematode Meloidogyne incognita in Response to Arabidopsis thaliana Root Signals
Source: PLoS One. 2013 Apr 12;8(4):e61259. doi: 10.1371/journal.pone.0061259 (PMC3625231; doi:10.1371/journal.pone.0061259)
Supplement: Table S1 — Transcript-derived fragments sequences. (DOCX) [file pone.0061259.s002.docx]

**Table S1: Transcript-derived fragments sequences**

| **>P11GH1**  ATGATGCTATTGTGGCTGAGACTTCTTCGTCAGGAAAGGAATGAAATGAATGCGGGAGCACCAAAGATGTATGCCCAAAATATTGGAGAACTTGGAAGTACAATGTTTCGATTCGGATAATTTACTCAGGACTCATCAATAATGGA |
| --- |
| **>P11GH2**  GAGGACAATTCCGTCAGGTGCAGACCCCAAAGACATGCTGGATTTACTCAGGACTCATCATCTCATGAT |
| **>P12AB2**  CTCCTCCTATCTACCAATTCTATTGTATTCACTCTCTCCATGTCTTGTTACTCAGGACTCATCCAAAA |
| **>P12AB4**  GATTTCTGCCCAGTGCTCTGAATGTTACTCAGGACTCATCAAA |
| **>P12CD3**  AGGTGTTCCATTATAATTGCTTCCCTCACTAAAGTTGGCAACATGGGTTTTCCCATTGTGTGTAAAATAAATTTCTTCAGCAGGCAAAGCTGTAACAGGAGATCTTCTATTACTGTTAGATATTTCCACTGCCTGACACCACAAGGTTACTCAGGACTCATCATG |
| **>P12CD5**  CACATCACAGGCCTCGTCGCAGCCAACTAAGCAAACCAGCAGCACTTCGAGTTTCCATATCGGTCAGAGTTCTCTGGACTACTGAT |
| **>P12CD6**  TGCCGGCGAGTGTTGATGCCTGCAATTTTTCAGCATCTTCATGGCCTGGGGGTATATCTTCATGATCGCTCATTAGGTTA |
| **>P12EF2**  TGCTACAAGCAATTGACAAGGAAGAGAAAGTAAACTTATAAAAGAGAATAATGGCCAAGTTGAATAAGTTGTATGTTGTTTGTTTTGTTTGTTGTTTTCGTTGTTGTTGTCGTTACTCAGGACTCATCAAAACAGCTGCTGAGGCGGAAGGA |
| **>P13AB1**  TTGGTATTCGTGCGTTGGAGCATTTGTTGGAGGTGCTCAATCTTACTCAGGACTCATCAGTTGCTCACGACTTGA |
| **>P13AB2**  GTGTTCGCCATGTTACAATGTAACGGTCATTCTTACTCAGGACTCATCACAACTT |
| **>P13AB3**  TGTTTATTATTTTTCGTCTTACTCAGAGACTCAACAAC |
| **>P13CD1**  ATCACACGTTTGTATAACCGAAGCTGGATCATGTGGTGAAATTGTTAGACAGCAGGATGATTATACTATTGTAAAAATGCCTACTCAACATGAATATGCGCTTACTCAGGACTCATCAAACGGTCA |
| **>P13GH1**  CCGTTAGGTTCTCATGTTGCTTAGAAGAAGACCGGACAGAAGTTTAGCATTATCAC |
| **>P13GH3**  ATTGGATGAGACACTTACCGAACTTACTCAGGACTCATCAC |
| **>P14EF2**  CAAGGTTCAGACGAGAAACGGACTCCTTATATCCAGATCCAGCACAAG |
| **>P14GH1**  GCACCTCCACTTTTTACCCAATGGTAAATGAATATTACAAATATACAAAAGACAAGGAATTTCTGAATGATATAATTCCTATGATGGAAAGGAGTTAGAATTTTGGACAGTCAATCGTTCTGTAGTGTTCGAAAAAGGCTGTAAAAGTTATACAATGTATCAATACAGAACAGAAAGTACCGTTCCAAGACCATAATCTTTTTGTGCAGACGTTATAACTGCAATTACTCGGGACTCA |
| **>P14GH2**  CAGCAGCAAGAATCTTTTCCTCAACACTATTAGCAGTAATTACTCAGGACTCATCAAGG |
| **>P14GH3**  AAATTTTCCGTCAAAGTGCATGTGCCGCCCAATTACTCAGGACTCATCAGCTCAA |
| **>P15CD1**  GGATAAATGCCAAAGTATTTCTTCTTTGGTGTTGTAACAGCCAAAGACTAAAAGTTCGGTCAGGACTCATCAAGAT |
| **>P15CD3**  CACCTCACTCACTAACCACTATACCATAAATAATCTTTTTTTGG |
| **>P16AB2**  ACACTTAACATAACAAATATAAGCAAAATCACAACCAGCTTCTCTTATACTTGTACCCAATTCTATATAACATAAAGAACCAAGAAAAGCAATTAAAGCACCAATAACCCAAACAAGGAGAGAAAGACCAGCAGATTCTGTATGTCGGTCAGGACTCATCCA |
| **>P16AB3**  CAGTTGTTGCTGCTGCTGTTGAGAATTGATATGTT |
| **>P16AB4**  GATTAGGACGAACATATTCCACAGTTAAAGCAGTATCACGGAAATGCAGAAGTGCATGATGCTGTGCACCTAAATTTCGTTGTTCACGCTGCTCTTGAACACGTTGTCGGTCAGGACTCATCAAATCACTG |
| **>P16AB6**  TTCAATATAAAATTTTTCTTTAACCCTATGCAATATCCGTAGGTGTTGTCGGTCAGGACTCATCCACAT |
| **>P16CD1**  TATGTACAACCGCATCCTCTTGCTGTTGTTGTTGTTGAAGAAGTAATTGTTGTTGTTGTTGATTTTGAGACATTTTTGACTTAGAACGTCGTGGTGCAGGCATCGGAGAAGAAATAGCAGCAGGTGTAGGGGCATTTCCAGGGTGAATTAAATGTTGCTGAATCTGTAATCTCTGTTGGAGCGATTGTTGAGAATGTGGCGATTGTGATATTTGCTGAGGTCCGACAGATGCTGGAGGTCGGTCAGGACTCA |
| **>P16CD2**  CTACCGTTTGTTGACGATAACACCGAGCGCGTGCTTGGTCACGTTGAAGACTTTGCCTGTTTTTCCATGATATGCTTTATGAGGCATTCCTTTCTGGAAAGCTCCATTTCCCTTAACAGTAACAATGTCTCCACGCTTATAAACTCTGTAAAATGTAGAAAGTGGCTCAGTTCCATGTCGACGGAAAGGTCGGTCAGGACTCAT |
| **>P16EF1**  GTATTCTACTGCTTTTACTTGCTTGTATGCCACCACGTCGGTCAGGACTCATACAA |
| **>P16GH1**  ATAAGGCTGCTCCTCCTATGGGCAATTATCTGTGAAAGGAAGTCAATTAGTGGGCAGTAATGGACAACCAGTTCAACTTGTTGGAATGTCACTTTTCTAGTCGGTCAGGACTCATACATC |
| **>P16GH4**  TTGGGAAGAAACACAATGAAGAACATTTGCAAATAAATCAAGTCGGTCAGGACTCATCAAAGCACT |
| **>P17AB1**  GACTGAGGGGGGTGGGGAATATACAAAAAGACATTTACCATTAAATAATCAACAACAACAAAGAATGCGTAAAA |
| **>P17AB3**  TCCATTTGCTATGACGCCATCATGATCAATGTCCATCCATTCTTTTATATATTGCAAAGAATATCCCCTCAACTCCGCATCGTCAACCTTTCCATTCCCATCAATATCCAGTTTTTTCAATTTTCCTTCAGTGAAGAAAGAGCGATATTTATTGATAGCCTCTTCGCTGTTCTCGGTCAGGACTCATC |
| **>P17AB4**  ATGCATCAATCATTTCATCCACCTCTCGTTTTGTTTCTGTCTCCATTTGGCTTTCATCATCGCCATCTTTCAATGTTTTTTCAACTTCAGTTAAAGCAGAAACAATTTGTTCGGCGCGTTTTTTAAAATGTGGAAGAACATTATCTCGGTCAGGACTCATC |
| **>P17AB5**  TAAACCTGTTTGCCATTTGATATTTTTGTTTGCGTCTCGGTCAGGACTCATCCAGCT |
| **>P17CD2**  TTGGGTTTGTTTCATATGATAATCCAAATTCTGCAACGACCGCTATTCAATCTATGAATGGTTTTCAAGTTGGAATGAAACGTTTGAAGGTGCAGCTGAAGAAACCTAGAGAGAAACCCTATTAGGTACGCAGTCTAAC |
| **>P17CD4**  GAGCAGTTGCACTATTTTTATAGCTCAATGAATGGAATGGAACTCGGATTGGCTCGGTCAGGACTCATCAGTTG |
| **>P17GH1**  TTATTCAGGAGGTCGCCCATCGCCACAACTCACTCATCCATCCCATTCACAACAACAGTCATCTTTTTTATCAATGGCTAGTGGCCCTCCACAAATCCATCAATTAGCACAACAACAGCAAGCTCCTTTCCATCAAATTGGAAACAATAATAGTAGTGGAGTTAATTATCAGCCTAAGCAATATGGCTCAACTGTTGGTGGCACAAACGCTTTTGACATGTCTCCACTTCTTTATGAGACTGGTATTGACAATAACACTCGGTCAGGACTCATCA |
| **>P17GH2**  TAGGAGGACGTGCCCCGTCCCGATGTCCATATCAGACCCATTAGAAGCCCCTAAAGTCTCATTTTCGTGGAACGACGAAGTACTCGGTCAGGACTCATCAA |
| **>P17GH3**  CATTCGTCCTCAAAGGTTGAACACTTCGCAAATAATTTCAAGCGATTCTGAGCCTGATATTGATCCGATGGGAAATTCATCACTAAATTTTCAGTCCTGTTATTCACATTTTATTAGTCGCTCTAATTCAAATACGGCAACTCGGTCAGGACTCATC |
| **>P49E1**  CAGGATGCTGAAAATGGAATGGGTTTACTCAGGACTCATCCATGA |
| **>P410A1**  ATGAGCCAAATTCTCTGCCGCTGCTTCAGGCTCTCTGTTCAACCCCGGTGGACAATGGGTCGAATCGTGCTTTACTCAGGACT |
| **>P412A1**  TTTGTTTTTGACTTTTTCACAAACTTTGTGTGCAAAATGACGCAAGCCTC |
| **>P412A2**  TGCTACGATCGTTACTCAAGACTCATCAGAGGAAAATAAAAATGGAAAAAGGGGGAGAGTTGACTTTATAATATCGCATTAGGTACGCAGTCTACACTAAA |
| **>P53E1**  TATTTACCAAATAAGCCGTCATCAGGGCATGTAATACCTGGAAATCGTGAAGATGATATTGGGATAGATTAC |
| **>P54E1**  TATCAATAGTTGGATTTTGCATAAGTCTTCTAATAAAGAAAATTGGTCGATCCTAA |
| **>P54E2**  GGCAATTGAACTCTTTTATTTTTTGACAAAAAATCCTTTTTTGACCCTCCAATTACTCAGGACTCATCAAGG |
| **>P54E4**  CCTAAGCTAGAATATATCCTAACAGAATATTACACCCCATCTTGGAGCAATTACTCAGGACTCATCACATCT |
| **>P55E1**  GACTTTTAAGAACAATATCCAATAAGTGCAGGGACCTTACATGCCTATAAAGCAGTAGAAATGCTTTATTGGTTACTTTCAAATTATGGACATGATAGAAATAATCCAGAAAGTAAAAATTATAATCAACGT |
| **>P57E2**  GACTAAAACGTTTCGGCGGATGGGGCGGTGGAACATACAGATATGGCCCTTGTGGTTACGGCGGATGTAATGGATATGGAAGTGGAGGAGGATGGATGAACATGGGAGCAGGCTATGGAATACGTCCAGTCGGTTGGCGACCATGGAGAACAGGTGGAGGAGGTCGTCATAGTCATGAAAGTCATAATAAAGAAAGTTATGGTTACTCAAGACTCAAC |
| **>P512A1**  AGGCTTTGCGGGAGGTTCAAGAGCACCTTCAATGGGAGCAGCTTCATTTGCATTACTCAGGACTCATCAAAGGGCCCAA |
| **>P61E1**  AGATTGTCATTGGTTGCCAAAAGTTGTTTTTGCCTTTACCATACTTTTTTTCTGTAATAATTTATCTCTCTGTTCCCGAAGATAATGCATTATTGATTGGATATTTTCATCGGGAATATCATCACGTTTTTTAAGTAAAGACCTATAAGCTAAATCGCTATTTTTATTTGAGTCCCCATCTTTTAACTTGTCTTTTCCACGTGGGGGCTGATGTACTTGACCTAAAGAACGGCGCTTTTTATTTGCACTTTCGGAAGAATTTTTGATGTTATTTTCGTCGTTTTTACTAGGTTTATCATTTCCACTTGTATCAGTTTCGTCATTTCTTTTTATCTCTTGTTTGTTCGGTCAGGACTCATCAAAA |
| **>P62A1**  AAGATTGTTTTTCACAATTTGGAGAATTACGTAAATCTGGCCATTTAACAGATGTTATAATTAAAGTTGAAGGAAGAGAAATTCCAGCACACAGAATTATTTTGGCTGCAACAATTCCTTTTTTTAATGCAATGTTTACTTGTGAAATGATTGAAGCTTATTCTAATGTTATTACTTTACCTAACATTGATGCAGACACAATGGAGTCAATTTTAAATTTTGCTTACTCTGGTCGGTCAGGA |
| **>P64A1**  ACGTAATACTTGCCATTCCAAGAGAGGAAGATGCTTAAC |
| **>P65E1**  TTATAGTGATCAAACACGATATCCTTTATCTGATCTGCCGCATTGTTTGACCGGGCTGTAAAAGACTGACGCAAATTATGAAGTTTCTTAGCCTCTTCTCCAGGGCGAACAGAACGTAAAAAATCGGCAACATATTCTGGTGCATTTAATTCTCCAGAAGTAAAAAATTCTGAATCATTGTTAACATTCTCCATAAGAATATCTGATAAATTACTAGACGAAAATACTTACTAAAATTTTGTTGTAAAATTTTTTGGAGAAGCAAATAAAAATTTAA |
| **>P66E1**  AACACATTCAGCCCACCCCGGAGGGTATATAGTTGCGGAGGTTAATGGACAGTCTGCGCAACTTGGATGTTTACAACGTTCTGTTTCAATTTTGTATTTGTAACAATCAGCTCTTTTCACTCGGTCAGGACTCATCAAGTGTGGATG |
| **>P67A1**  TCCTGTAGCTATAATCGTTGCTCACACAACA |
| **>P67E1**  TGGCCAAACGCCGAATTGAAAAATCACACATGATAACACGAGGAAATAGTTTAGATTCCCCCCAATCCACACCTTTGAACAAGTCAAAAACGGCCTGCCAAGCCCAACTATGAA |
| **>P69A1**  TACCTGCATACCGTTGGATATAGTCCGAGAGACTCAAATCACATAATTCTAAGG |
| **>P611E1**  AGAGGAGCTGGCAGTTGAAGGCAAATTTGTAATAATTCCTGGCTTGGCTCTGCGAACGTAAAGCATGGCGACGTTCTCGTGTACGAGAACGGGAACGTTCTAACAATCGTGAAGTACAATAAAGGGAAGAAGCGAATGGACCACTAGTCACTAAATCATCAAAGAAGGCGGTGTAGGCTGCTGGTGCTGCTGGCGGTTCACCAACTGTGTACTCATTACTATATTTGCTGGAGCGGTCGGTG |
| **>P611E3**  GACTGTCACTTTTCCGTCCTGACATTTTGTTTTTGGTCACAGTGGTGCT |
| **>P612E2**  AATGCGTACAGGGGGCTCATTTGGTGTATTATCAACGGAGGAAAAGGCAGCAAGTCGGTCAGGACTCATCAAAG |
| **>P78E1**  AATAACGAACCTTTAATAAATCTGCAGATTATTCAATGATGCTCTTCCTCTTCCCCCTCCCTTATTATTAAGTGCTGTAAACATAAAGGATGACCAACATATTCAACGGTTTGGCAATGAGCATGATGATAGTTTTCCACTTCCCCCGCCTCCATTAACAGTCGGTCAGGACTCATC |
| **>P79E1**  CTCTGTCACAACAAACAACTTTATTTACTTTTGGGGCATTCGCGATTTCTCTGCCTTTTTCGCTTCTTTCTTGTGAATCTTCTCCCATTTGGCTGCCATTTTTGAAGCATGGGCTTCCGCTATTTGAACGTCATCCTTTTCCGCGTTGTTTGTGGAGGCTGCTGCTTCGGCCTGCTCTTGTTCCAATTCTTTCTGGGCTCTCGCTCTCTCCTCTTTGAGTTGCTTGAATTCTTCCTCCATTTGTTTCCTCTTCTCTTCGCTCTTCTCGCTCGGTCAAGACTCATCAA |
| **>P79E2**  CGAATTTTTGGTTCCACGATGAGTAGATCCAATACGATCACGTTCGTTTTGTATTGCTTCAATCCTCATTCCTTCAATCAAACATTTTTT |
