# Supplementary material for: Transcriptional Changes of the Root-Knot Nematode Meloidogyne incognita in Response to Arabidopsis thaliana Root Signals
Source: PLoS One. 2013 Apr 12;8(4):e61259. doi: 10.1371/journal.pone.0061259 (PMC3625231; doi:10.1371/journal.pone.0061259)
Supplement: Table S2 — Summary of the 63 transcript-derived fragments (TDF)a. a Differential expression between ARE- and H2O-treated Meloidogyne incognita infective juveniles b Presence (Y) or absence (N) of signal peptide (SP) was determined by using SignalP software. When no full cDNA or protein sequence was available, the presence of a SP was not determined (Na). c Putative localisation of the protein corresponding to the TDF was analysed using WoLF PSORT software. Only 1 predicted localisation was reported when the score was superior at 18. d Proteins were classified by family using the classification published in Bellafiore et al., 2008. (1 = proteins interacting with actin/microtubules, 2 = proteins interacting with nucleic acids, 3 = post-translational modifications, protein turnover, and chaperone functions, 4 = metabolism, 5 = signal transduction, 6 = proteins synthesis and secretion, 7 = detoxification, 8 = cell wall modification enzymes, 9 = others) e Gene expression is reported for ARE-treated nematodes in comparison to H2O-treated nematodes (2 biological repeats). (DOCX) [file pone.0061259.s003.docx]

**Table S2: Summary of the 63 transcript-derived fragments (TDF)^a^**

| TDF  name | *M. incognita* genome or NCBI accession no. | *M. incognita* genome or NCBI annotation | Highest homology in BLASTP or BLASTX | BLASTP or BLASTX  E value | Signal peptide^b^ | predicted localization WoLF PSORT^c^ | Protein class^d^ | Gene expression normalised^e^ |
| --- | --- | --- | --- | --- | --- | --- | --- | --- |
| **P11GH1** | RN0AAA90YJ17RM1 | Na | No homology |  | N | Na | 9 | 0.77 |
| **P11GH2** | *Minc02836*  *Minc04761 Minc04747* | Not annotated | No homology |  | N | Extracellular | 9 | 1.42 1.45 1.18 |
| **P12AB2** | *Minc11004* | Phospho-fructokinase | 6-phosphofructo-2-kinase/fructose-2,6-biphosphatase [Ascaris suum ] | 0.E+00 | N | Mitonchondria | 4 | 1.04 |
| **P12AB4** | *Minc14272* | inositol polyphosphate 4 phosphatase | Type I inositol-3,4-bisphosphate 4-phosphatase [Ascaris suum] | 1.E-173 | N | Cytoplasmic | 4 | 1.04 |
| **P12CD3** | *Minc14575* | Immunoglobulin subtype; Myosin Light Chain Kinase | 2 (Zwei) IG-domain protein family member (zig-1) [Caenorhabditis elegans] | 8.E-25 | Y | Plasma membrane/ER | 9 | 0.95 |
| **P12CD5** | *Minc14134* | Patatin | Unknown  [Ascaris suum] | 3e-100 | Y | nucl: 7.0, E.R._mito: 6.5, extr: 6.0, mito: 6.0, cyto: 5.5, cyto_pero: 5.0, E.R.: 3.0 | 9 | ND |
| **P12CD6** | *Minc13031* | EF-Hand type; Ryanodine receptor-related | Ryanodine receptor 44F [Ascaris suum] | 0.E+00 | N | Plasma membrane | 5 | 1.24 |
| **P12EF2^1^** | AW827744 | Na | HEAT repeat-containing protein [Dictyostelium discoideum AX4] | 2.E-02 | N | nucl: 13.0, mito: 11.0 |  | 1.18 |
| **P13AB1** | *Minc14728* | Phosphofructokinase; Ribosomal protein S2 | 6-phosphofructokinase [Ascaris suum] | 0.E+00 | N | Cytoplasmic | 4 | 1.27 |
| **P13AB2** | *Minc04338* | Translation initiation factor IF2/IF5; eIF4-gamma/eIF5/eIF2-epsilon | Eukaryotic translation initiation factor 5 [Ascaris suum] | 1.E-126 | N | cyto: 12.5, cyto_nucl: 11.0, mito: 9.0, nucl: 8.5 | 6 | 1.1 |
| **P13AB3** | *Minc09676* | Malate synthase-like, core | hypothetical protein C10F3.4 [Caenorhabditis elegans] | 6E-53 | N | cyto: 9.0, nucl: 8.0, plas: 5.0, E.R.: 3.0, mito_pero: 3.0, extr: 2.0 | 4 | 1.01 |
| **P13CD1** | *Minc08365* | Ribosomal protein L2; Translation protein SH3-like | 39S ribosomal protein L2 [Ascaris suum] | 4.E-98 | N | Mitochondria | 6 | 1.04 |
| **P13GH1** | RN0AAA193YP05FM1 | Na | hypothetical protein LOAG_08817 [Loa loa] | 3e-11 | Na | Na | 9 | 2.81 |
| **P13GH3** | *Minc10328a Minc06691a* | Myosin S1 fragment, N-terminal; Prefoldin; Heavy chain of Myosin | Myosin tail family protein  [Brugia malayi] | 0.E+00 | N | cyto_nucl: 17.3, nucl: 17.0, cyto: 13.5, cyto_mito: 7.8 | 1 | 1.41 1.10 |
| **P14EF2** | *Minc01905* | Proteasome/cyclosome, regulatory subunit | 26S proteasome non-ATPase regulatory subunit 1  [Ascaris suum] | 0.E+00 | N | cyto: 16.5, cyto_nucl: 15.5, nucl: 13.5 | 3 | 0.98 |
| **P14GH1** | *Minc10188* | Glycoside hydrolase, family 37 | Trehalase family protein [Brugia malayi] | 3.E-123 | Y | Extracellular | 4 | 1.34 |
| **P14GH2** | *Minc17982* | SNF2-related; Bromodomain; DNA/RNA helicase | BRM protein [Brugia malayi] | 0.E+00 | N | Nuclear | 2 | 1.19 |
| **P14GH3** | *Minc05259* | Not annotated | hypothetical protein [Culex quinquefasciatus] | 1.E-15 | N | cyto: 16.0, cyto_nucl: 15.5, nucl: 13.0 | 9 | 1.07 |
| **P15CD1** | *Minc16284* | Not annotated | hypothetical protein F33D11.5 [Caenorhabditis elegans] | 4.E-11 | N | cyto: 14.5, cyto_nucl: 11.0, mito: 8.0, nucl: 4.5, extr: 2.0 | 4 | 1.33 |
| **P15CD3** | MiV1ctg1047:1712..1684 | Na | No homology | Na | Na | Na | 9 | 0.77 |
| **P16AB2** | MiV1ctg1196:15..1056 | Na | CRE-AAT-5 protein [Caenorhabditis remanei] | 2.E-75 | Na | Na | 5 | 1.16 |
| **P16AB3** | MiV1ctg2211:3242..5562 | Na | CRE-SCD-1 protein [Caenorhabditis remanei] | 2.E-17 | Na | Na | 9 | ND |
| **P16AB4** | *Minc07561* | E3 ubiquitin ligase | E3 ubiquitin-protein ligase MARCH6 [Ascaris suum] | 7.E-47 | N | Cytoplasmic | 3 | ND |
| **P16AB6** | *Minc03819* | Ovarian tumour, otubain | Ubiquitin thioesterase otubain-like protein [Ascaris suum] | 1.E-80 | N | Plasma membrane | 3 | 0.84 |
| **P16CD1** | *Minc16757* | Homeodomain protein CUT | C. briggsae CBR-CEH-38 protein [Caenorhabditis briggsae] | 1.E-88 | N | Nuclear | 2 | 1.30 |
| **P16CD2** | *Minc04060* | Ribosomal protein L21e | 60S ribosomal protein L21 [Ascaris suum] | 2.E-60 | N | Nuclear | 6 | 1.01 |
| **P16EF1** | MiV1ctg1025:1..778 | Na | hypothetical protein DDB_G0280555 [Dictyostelium discoideum AX4] | 3.E-02 | Na | Na | 9 | 0.52 |
| **P16GH1** | *Minc13221 Minc09298 Minc09446* | Glycoside hydrolase, family 5; Carbohydrate-binding | beta-1,4-endoglucanase [Meloidogyne incognita] | 0.E+00 | Y | Extracellular | 8 | 1.45 1.29 ND |
| **P16GH4** | *Minc09245* | Transaldolase; Isocitrate/isopropylmalate dehydrogenase | Transaldolase [Ascaris suum] | 7.E-121 | N | cyto: 13.0, cyto_nucl: 10.5, cysk: 8.0, nucl: 6.0, mito: 2.0 | 4 | 0.93 |
| **P17AB1** | RN0AAA101YO11RM1 | Na | No homology | Na | Na | Na | 9 | 0.98 |
| **P17AB3** | *Minc00672* | EF-Hand type | calmodulin, putative [Magnaporthe oryzae 70-15] | 7.E-04 | Y | Extracellular | 5 | 0.83 |
| **P17AB4** | *Minc06992* | Not annotated | pre-mRNA-splicing ATP-dependent RNA helicase prp28, putative [Pediculus humanus corporis] | 1.E-42 | N | Nuclear | 2 | 1.16 |
| **P17AB5** | *Minc02608* | Nucleic acid-binding, OB-fold | Replication factor A 73 kDa subunit [Ascaris suum] | 1.E-76 | N | Nuclear | 2 | ND |
| **P17CD2** | *Minc16232* | Paraneoplastic encephalomyelitis antigen; Nucleotide-binding | tnrc4 protein [Xenopus (Silurana) tropicalis] | 7.E-102 | N | Nuclear | 2 | ND |
| **P17CD4** | *Minc16473* | Sodium dicarboxylate symporter | Excitatory amino acid transporter [Ascaris suum] | 9.E-154 | N | Plasma membrane | 5 | 1.11 |
| **P17GH1** | MiV1ctg2132:4969..5166 | Na | No homology |  |  |  | 9 | 1.14 |
| **P17GH2** | BM774089 | Protein tyrosine phosphatase precursor | Receptor-type tyrosine-protein phosphatase-like protein  [Ascaris suum] | 8.E-36 | Na | Na | 5 | 1.11 |
| **P17GH3** | *Minc04919* | ATG2, C-terminal | Autophagy-related protein 2 B  [Ascaris suum] | 1.E-146 | N | Plasma membrane | 3 | 1.26 |
| **P49E1** | *Minc15563* | Ankyrin | protein C33A11.1 [Brugia malayi] | 4.E-77 | N | Extracellular | 1 | 1.27 |
| **P410A1** | MiV1ctg1152:7776..9917 | Na | EXPB1 protein [Globodera rostochiensis] | 3.E-02 | Na | Na | 8 | 0.87 |
| **P412A1** | MiV1ctg2262:5162..5209 | Na | Transcription factor Dp-1 [Ascaris suum] | 4e-27 | Na | Na | 2 | 1.20 |
| **P412A2** | *Minc12241* | Voltage-dependent calcium channel | Calcium Channel, Beta subunit family member (ccb-1) [Caenorhabditis elegans] | 0.E+00 | N | Nuclear | 5 | 1.14 |
| **P53E1** | [BM882988](http://www.ncbi.nlm.nih.gov/entrez/query.fcgi?cmd=Retrieve&db=Nucleotide&list_uids=19266732&dopt=GenBank) | Not annotated | hypothetical protein, conserved in Plasmodium species [Plasmodium knowlesi strain H] | 2.E+00 | Na | Na | 9 | 0.99 |
| **P54E1** | *Minc18470* | Not annotated | hypothetical protein C17A2.4 [Caenorhabditis elegans] | 8.E-15 | N | Cytoplasmic | 9 | 1.03 |
| **P54E2** | *Minc03950* | Not annotated | Hypothetical protein CBG17891 [Caenorhabditis briggsae] | 9.E-06 | Y | Extracellular | 9 | 0.94 |
| **P54E4** | *Minc01998a* | Not annotated | No homology |  | Y | Extracellular | 9 | 1.29 |
| **P55E1** | *Minc17848* | ADP-specific phosphofructokinase/glucokinase | ADP-specific Phosphofructokinase/Glucokinase [Brugia malayi] | 1.E-27 | N | nucl: 13.0, cyto: 11.0, extr: 5.0 | 4 | 1.16 |
| **P57E2** | *Minc18288* | Not annotated | No homology |  | Y | Extracellular | 9 | 2.89 |
| **P512A1** | *Minc14735* | Not annotated | CRE-TNT-2 protein [Caenorhabditis remanei] | 1.E-98 | N | nucl: 19.0, cyto_nucl: 15.3, cyto: 9.5, cyto_mito: 5.7 | 1 | 1.27 |
| **P61E1** | RN0AAA457YE15RM1 | Na | No homology |  |  |  | 9 | 1.38 |
| **P62A1** | *Minc08550* | Galactose oxidase/kelch, beta-propeller | Kelch-like protein 18 [Ascaris suum] | 3.E-148 | N | nucl: 16.5, cyto_nucl: 16.0, cyto: 14.5 | 4 | 1.28 |
| **P64A1** | AW828322 | Diamine acetyltransferase | hypothetical protein CRE_27333 [Caenorhabditis remanei] | 1.E-18 | N | cyto: 17.5, cyto_nucl: 13.5, nucl: 6.5, cysk: 4.0 | 4 | 1.87 |
| **P65E1** | *Minc03640 Minc15719* | Not annotated | CRE-EXOC-8 protein [Caenorhabditis remanei] | 3.E-07 | N | cyto_nucl: 17.5, cyto: 16.0, nucl: 9.0, cysk: 4.0 | 1 | 1.31 |
| **P66E1** | *Minc04733* | Not annotated | No Homology |  | N | Extracellular | 9 | 1.66 |
| **P67A1** | *Minc11065* | Excinuclease ABC, B subunit; Rh-like protein/ammonium transporter | vasa homolog [Ciona savignyi] | 2.E-53 | N | Nuclear | 2 | ND |
| **P67E1** | *Minc03595* | Innexin | Innexin family protein [Brugia malayi] | 7.E-37 | N | mito: 12.0, extr: 7.5, golg: 6.0, extr_plas: 4.5, E.R.: 4.0 | 1 | ND |
| **P69A1** | *Minc09681* | Annexin | Annexin-B11 [Ascaris suum] | 2.E-129 | N | cyto: 14.5, cyto_nucl: 13.0, nucl: 10.5, pero: 3.0 | 5 | 1.10 |
| **P611E1** | *Minc18706* | Not annotated | hypothetical protein CRE_03298 [Caenorhabditis remanei] | 1.E-66 | N | Nuclear | 9 | 1.31 |
| **P611E3** | *Minc11760* | Tubulin; Beta tubulin, autoregulation binding site | beta-tubulin [Haemonchus contortus, Peptide, 448 aa] | 0.E+00 | N | cyto: 10.0, cyto_nucl: 10.0, nucl: 8.0, pero: 8.0, extr: 3.0 | 1 | 1.08 |
| **P612E2** | *Minc00911* | Not annotated | Hypothetical protein CBG06665 [Caenorhabditis briggsae] | 4.E-50 | N | Plasma membrane | 9 | 0.92 |
| **P78E1** | *Minc09250* | general substrate transporter | Solute carrier family 2 [Ascaris suum] | 1.E-24 | N | Plasma membrane | 5 | 1.18 |
| **P79E1** | *Minc08375* | Zinc finger, LIM-type | LIM domain protein variant [Cyathostominae sp. JM-2007a] | 4.E-107 | Na | Na | 2 | 1.09 |
| **P79E2** | *Minc11307* | Vitamin D receptor; Zinc finger, NHR/GATA-type | Nuclear hormone receptor family member nhr-14 [Ascaris suum] | 1.E-65 | N | Nuclear | 5 | 1.18 |

^a^ Differential expression between ARE- and H_2_O-treated *Meloidogyne incognita* infective juveniles

^b^ Presence (Y) or absence (N) of signal peptide (SP) was determined by using SignalP software. When no full cDNA or protein sequence was available, the presence of a SP was not determined (Na).

^c^ Putative localisation of the protein corresponding to the TDF was analysed using WoLF PSORT software. Only 1 predicted localisation was reported when the score was superior at 18.

^d^ Proteins were classified by family using the classification published in Bellafiore et al., 2008. (1= proteins interacting with actin/microtubules, 2= proteins interacting with nucleic acids, 3= post-translational modifications, protein turnover, and chaperone functions, 4= metabolism, 5= signal transduction, 6= proteins synthesis and secretion, 7=detoxification, 8= cell wall modification enzymes, 9= others)

^e^ Gene expression is reported for ARE-treated nematodes in comparison to H_2_O-treated nematodes (2 biological repeats).
