# Supplementary material for: Transcriptional Changes of the Root-Knot Nematode Meloidogyne incognita in Response to Arabidopsis thaliana Root Signals
Source: PLoS One. 2013 Apr 12;8(4):e61259. doi: 10.1371/journal.pone.0061259 (PMC3625231; doi:10.1371/journal.pone.0061259)
Supplement: Table S3 — Oligonucleotides used for qRT-PCR. (DOCX) [file pone.0061259.s004.docx]

**Table S3: Oligonucleotides used for qRT-PCR**

| TDFs | Forward primer | Reverse primer |
| --- | --- | --- |
| **P11GH1** | GTTTAATAACCAAGCATTGG | CCAAGTTCACCAATATTTTG |
| **P11GH2** | TGGAACTACCGAAGGAGGATTGGAA | CCCTCCAAATCCTCTTTCCAAACCT(MINC02836)  TCGTCCACCATTCGGTAAATCTCG (MINC04761)  CTCGTCTTCCCTCTCCTTCTTTTCCA (MINC04747) |
| **P12AB2** | GCGGAAGTCAAAATAAGTTCTCCCG | CGACTTTGGACGTGGCCATTTACG |
| **P12AB4** | GGAATTTGTTGGGGGAGATTGTGTG | CCTCCATTAGAATACCGCAAAGGCA |
| **P12CD3** | CAACAAAAACAGAGGGCAACAACCA | CCATTGCCCTGCATGATCAACATAA |
| **P12CD5** | TACGGTGAGCCCTATGTTCGGC | GCAAATTGCATTAATTCAAAGGGCA |
| **P12CD6** | TGGTCAGCAATCAAAGAAAACGTGC | GGCACATAATTGCTGCACCCCTATT |
| **P12EF2** | CTTTTGCTCAAGATGAAGATT | CAAGCAATTGACAAGGAAGA |
| **P13AB1** | ATGTATGGAGCCTGGATGCTTTGC | GTTGAAGGGCAGTATCCGTTCCAAT |
| **P13AB2** | GCCTTTTGGCCAAAGTTGAGGGAAA | GGATTGTCACATGCTGGGCACAA |
| **P13AB3** | TCTTACCGACTGCTCTGAGGAACGA | CGGGGGAATGTTGAGATGAAATTG |
| **P13CD1** | CTGGAAGAAAGTCCAATATTCACAT | CCAACAGTTGCCATGCAAGTCTTTT |
| **P13GH1** | TGGAAAGGGCAGAGGCTGAATTATT | CCCAGGCCGAAAAAGAATTGTAAGA |
| **P13GH3** | AATACAACGAGAGCTCGAGGATGCC | TGGCGAGTCTTCTCCAGAGATTGAA(MINC10328)  CCATGAGTTTGTGGCGAGTCTTGC (MINC06691) |
| **P14EF2** | ATGGCGCAAGTTATGCATATTTGGA | GCGTCGTTCCAAGTTTTGTTGAAAA |
| **P14GH1** | TATTTTCCCTCCCCTCCTCTTTTGG | GGGTTTTGGTTGGAAGTTTGGAATG |
| **P14GH2** | CAAGACGCAAAGCACGAAGTTCAAT | CCCAGACAATTATTGAACCAAGCGA |
| **P14GH3** | TTTGGCATAACGGATTTCGCTATCA | CGTTTGGGCGAACTTTTCCATAAA |
| **P15CD1** | TTAAATGCTGATGGAGCTTTTGGGG | CATCAGCAAATTTAAGCGCCTCAAA |
| **P15CD3** | GAGGGTTCGAGGCCCATTGAGGTTA | GGGGACTCGAACCTACGACCATTCT |
| **P16AB2** | TATAATCCCCCCACCATCCCCTACA | GAGGGAAAGACCGGCAGATTCTGTA |
| **P16AB3** | TAATGTCGGACAAAATGTTTCGGCA | TGTTTAACTCTTGTCCGGCTTGTGC |
| **P16AB4** | CGAACGCATCGACTGGTATTCTCC | GCAGGTTCGTCATCCGTTGTGTCAT |
| **P16AB6** | ATTGCTCTAGTCAATGCGATTGGGG | GGATCCCAGGGCATAAAAGCAAATA |
| **P16CD1** | TGGATATTCATTAGGCCAACCGACC | TGCCTGAGGGCATTGGACCTTTATA |
| **P16CD2** | ATATGTTTGCTCGTCCTTTCCGTCG | GCGATTCCTAACACGTTTGTTGACG |
| **P16EF1** | TAATTATTTGCCGTCGACGTGGTGG | TGAAATGCAAAGGCGTTTGGTAAGA |
| **P16GH1** | GTAAGCGGCACAAACCTTTGC | TTGACGATGCACCACTGCAACTGACACCG(MINC09446)  CTGGCTCCGGCATTGCTACCCGTA(MINC13221)  TTTGCCCCAGCATTGCTGCCCGTA(MINC09298) |
| **P16GH4** | GCTTGTGCTCAGGCAAAGGTTACTCT | CAAAAGATCACAACCAGCCAATCCTTT |
| **P17AB1** | TCTTCAAAATGAATGCCGTTATCCG | ACCCGTTCCAGTATGTTCCACCATT |
| **P17AB3** | GAAGGCAAACTTGTTTACGGCAGC | GACGCCATCATGATCAATGTCCATC |
| **P17AB4** | GCAAGCCAAAATTTTTGACAAAAGCTG | TGCATTCTTTCCCTATCCTTTTCAGCT |
| **P17AB5** | TCTTTGCTTGAAACGTCCGCTGA | GCGTCTCGAGTCGAAGCAAAATATG |
| **P17CD2** | ATATGTATTTGGCACCTTCGTGCGC | CCCACCTTCAAACGTTTCATTCCAA |
| **P17CD4** | TGTGGTATTCACCAATTGGCATTTCTT | TCCATATTAATTGTTGCCCCAACAGGT |
| **P17GH1** | ATTCAGGAGGTCGCCCATCG | CAAAAGCGTTTGTGCCACCAACAG |
| **P17GH2** | ACCATTTTCATTCGTTGATCACGCA | TTTGGAGCGAGCATTACGTCATCC |
| **P17GH3** | CATTTTTATGATTTCCATTCGGGCG | CGTGTCTGTTGTTCCCCTGGTTTAA |
| **P49E1** | CCGATTTAATGTTTGGCGAAGAGCA | GAAAGGTTGAGGTGCACCATGAACA |
| **P410A1** | TTGCCCTTATATTTCCAGACCACCC | CCACGTCCACCAGTTCCATAAAATG |
| **P412A1** | CAACAGACAAAACTAGGGGCTTGCG | ACGTCGGCGAATATTACGAACATCA |
| **P412A2** | TGGGTGCTTTAGACAGAATGATGCC | CCAACAAGCACTACAGGCCTCATTG |
| **P53E1** | GCTTTGCTTTAATGCAGGTTGATGC | CCAGGTATTACATGCCCATTTGACG |
| **P54E1** | TTAGATTCGATGAGAGAAAGGCCGG | GCAGCAGTAACCAAGAATGAATCGC |
| **P54E2** | ATGCAGCAAACAGGCCCTTTCC | TGCACGTTTAGCGAGCATTTGTTG |
| **P54E4** | GCTTTTGGGATTTGTTTTGGCATTG | CTTATTGTGACATTCGCAGGGCCT |
| **P55E1** | TGCCTATAAAAATGGCAGTAGAAATGC | GCCAAATTACAAGATTGACGTCCAGCT |
| **P57E2** | GGCTAAAACGTTTTGGCGGATGGG | TCCTCCGCCTGTTCTCCATGGT |
| **P512A1** | TATTCCCGAAAAGGGTGCTGGTGGT | TCGGGTCAACACCAGTATTCAAAGC |
| **P61E1** | GATTGTCATTGGTTGCCAAAAGTTGTT | AAAAAGCGCCGTTCTTTAGGTCAAGTA |
| **P62A1** | TATTCCTCAAGCCGTAATCGATGCC | AAGGTAGGCAGGCCGTAAAAGAGGT |
| **P64A1** | AATTCGATTGGCGAAGCCTGATGAT | CCAATTCCCCGTCGTCGAAATT |
| **P65E1** | TTTATACTTGCGTTGCGAAAATGCC | CATCCGTCTTCGTTTTCCAAGTTCA |
| **P66E1** | CAAAGCAAAATATTCGAGTGAAAAGAGCAG | TTATCCATCATAGTAACAACATTCAGCCCA |
| **P67A1** | GGACTGGCCGCATAGGAACAGAA | CCACTTTCCAGTAATCCAAAACCCG |
| **P67E1** | CGCGAACTCCATCGATTTGTCC | GGATTGGCCAAACGTCGAATTGAA |
| **P69A1** | GTCTGCTTACAACCAACCAGCTGGG | CGTAGTAACTCAGCCGTCTGTTGCG |
| **P611E1** | GACCGCGATTCATACAAGCCAATT | GGGGAGCGAAACCTATTTGACCAC |
| **P611E3** | GGTCAATGTGGAAACCAAATCGGG | TGCCAGCACCACTCTGACCAAAA |
| **P612E2** | TGCCTGGTATTTATTGCCTCCTCCA | TGCATTGGATCTTGTAGCGATGACA |
| **P78E1** | CAAATTTATGGTGATGGGCAACACG | GGGGAAGAGGAAGAGCATCATCTGA |
| **P79E1** | ATGGGAGATTGGTAAGTGGGAAGGG | GGCCAAATTTTCCTTTCCAACACCT |
| **P79E2** | AAGGTGAAGTTGGAATGGGAGATGG | CAGGCATTCCGACGAGTTTTGTCTA |
| ***MAP-1*** | TCGGCTTTTTGGCCATTTCTACATC | GCGATTGCAAACACGTCCACAACTT |
| **Actin** | GATGGCTACAGCTGCTTCGT | GGACAGTGTTGGCGTAAAGG |
| **EF1** | TGGGAGTCAAGCAACTTATCG | GTCAAGGGCTTCCAACAAAGT |
| **GPDH** | AGCAATGGAAATTTGGTAGTTGA | GGAGCTGAGATAACCACCTTCTT |
