# Supplementary material for: Transcriptional Changes of the Root-Knot Nematode Meloidogyne incognita in Response to Arabidopsis thaliana Root Signals
Source: PLoS One. 2013 Apr 12;8(4):e61259. doi: 10.1371/journal.pone.0061259 (PMC3625231; doi:10.1371/journal.pone.0061259)
Supplement: Table S4 — Mean ΔCT for statistical comparison. LSD = Least significant difference, degree of freedom (df) = 19 except for P16EF1 and P17AB3 (df = 18) (DOCX) [file pone.0061259.s005.docx]

**Table S4: Mean ∆CT for statistical comparison.**

| **Gene** | **Time in h** | | | | | | | | | | | **LSD** |
| --- | --- | --- | --- | --- | --- | --- | --- | --- | --- | --- | --- | --- |
|  | **0** | **2** | **4** | **6** | **8** | **12** | **24** | **48** | **72** | **96** | **168** |  |
| **P16EF1** | 7.79 | 6.03 | 5.78 | 5.22 | 5.73 | 5.93 | 6.36 | 4.61 | 4.07 | 3.91 | -0.02 | 2.103 |
| **P57E2** | 8.25 | 1.69 | 3.02 | 3.49 | 3.78 | 4.91 | 5.32 | 3.17 | 2.99 | 2.42 | -0.29 | 1.991 |
| **P17AB3** | 10.86 | 7.35 | 12.53 | 7.94 | 7.12 | 7.05 | 6.16 | 5.58 | 5.49 | 6.43 | 2.09 | 2.698 |
| **P66E1** | 9.09 | 4.86 | 6.65 | 6.13 | 7.83 | 7.32 | 8.07 | 6.19 | 5.19 | 4.13 | 0.64 | 2.478 |
| **P16AB6** | 8.42 | 9.95 | 13.52 | 10.13 | 7.97 | 8.53 | 6.93 | 7.48 | 4.87 | 4.1 | 3.35 | 1.942 |
| **P64A1** | 7.61 | 6.49 | 7.8 | 7.5 | 8.13 | 7.93 | 7.47 | 5.94 | 5.48 | 4.91 | 2.74 | 1.679 |
| ***MAP-1*** | 11.56 | 6.71 | 7.02 | 5.68 | 5.15 | 4.27 | 3.72 | 3.35 | 2.24 | 1.66 | 5.01 | 1.655 |

LSD = Least significant difference, degree of freedom (df) = 19 except for P16EF1 and P17AB3 (df = 18)
